# RNA Seq analysis of the *Eimeria tenella* gametocyte transcriptome reveals clues about the molecular basis for sexual reproduction and oocyst biogenesis

**DOI:** 10.1186/s12864-015-1298-6

**Published:** 2015-02-18

**Authors:** Robert A Walker, Philippa A Sharman, Catherine M Miller, Christoph Lippuner, Michal Okoniewski, Ramon M Eichenberger, Chandra Ramakrishnan, Fabien Brossier, Peter Deplazes, Adrian B Hehl, Nicholas C Smith

**Affiliations:** Queensland Tropical Health Alliance Research Laboratory, Australian Institute of Tropical Health and Medicine, James Cook University, Cairns Campus, McGregor Road, Smithfield, QLD 4878 Australia; Institute of Parasitology, University of Zurich, Winterthurerstrasse 266a, CH-8057 Zürich, Switzerland; College of Public Health, Medical and Veterinary Sciences, James Cook University, Cairns Campus, McGregor Road, Smithfield, QLD 4878 Australia; Department of Farm Animal, University of Zurich, Winterthurerstrasse, CH-8057 Zürich, Switzerland; Functional Genomics Center Zurich, Winterthurerstrasse, CH-8057 Zürich, Switzerland; Apicomplexes et Immunité Mucosale, INRA, UMR1282, Infectiologie et Santé Publique, F-37380 Nouzilly, France; Université François Rabelais de Tours, UMR1282, Infectiologie et Santé Publique, F-37000 Tours, France

**Keywords:** *Eimeria tenella*, RNA Seq, Microgametocyte, Macrogametocyte, Oocyst, Fertilisation, Transmission

## Abstract

**Background:**

The protozoan *Eimeria tenella* is a common parasite of chickens, causing avian coccidiosis, a disease of on-going concern to agricultural industries. The high prevalence of *E. tenella* can be attributed to the resilient oocyst stage, which is transmitted between hosts in the environment. As in related Coccidia, development of the eimerian oocyst appears to be dependent on completion of the parasite’s sexual cycle. RNA Seq transcriptome profiling offers insights into the mechanisms governing the biology of *E. tenella* sexual stages (gametocytes) and the potential to identify targets for blocking parasite transmission.

**Results:**

Comparisons between the sequenced transcriptomes of *E. tenella* gametocytes and two asexual developmental stages, merozoites and sporozoites, revealed upregulated gametocyte transcription of 863 genes. Many of these genes code for proteins involved in coccidian sexual biology, such as oocyst wall biosynthesis and fertilisation, and some of these were characterised in more depth. Thus, macrogametocyte-specific expression and localisation was confirmed for two proteins destined for incorporation into the oocyst wall, as well as for a subtilisin protease and an oxidoreductase. Homologues of an oocyst wall protein and oxidoreductase were found in the related coccidian, *Toxoplasma gondii*, and shown to be macrogametocyte-specific. In addition, a microgametocyte gamete fusion protein, EtHAP2, was discovered.

**Conclusions:**

The need for novel vaccine candidates capable of controlling coccidiosis is rising and this panel of gametocyte targets represents an invaluable resource for development of future strategies to interrupt parasite transmission, not just in *Eimeria* but in other Coccidia, including *Toxoplasma*, where transmission blocking is a relatively unexplored strategy.

**Electronic supplementary material:**

The online version of this article (doi:10.1186/s12864-015-1298-6) contains supplementary material, which is available to authorized users.

## Background

*Eimeria tenella* is an obligate intracellular protozoan parasite of the phylum Apicomplexa and one of the main causes of avian coccidiosis. Diarrhoea, anaemia and mortality are the main manifestations of clinical coccidiosis but in sub-clinical infections, ineffective feed conversion due to malabsorption is the most important consequence. Coccidiosis is estimated to cost poultry industries around the world in excess of US $3 billion per year [1,2]. With the development of drug resistance in *Eimeria* species threatening the continued use of prophylactic anticoccidials, vaccination remains a desirable long-term strategy for combatting this disease [3].

The lifecycle of *E. tenella* is, arguably, the least complex of all Coccidia and serves as a model to understand the lifecycles of many other important parasites in this group. *Toxoplasma gondii,* for instance, has a particularly complex lifecycle and is able to infect a variety of intermediate hosts but reproduces sexually only in felids (reviewed in [4]). In *E. tenella,* following ingestion of a sporulated oocyst, released sporozoites migrate to the intestinal epithelia and undergo three rounds of asexual reproduction, producing successive generations of merozoites. Third-generation merozoites then differentiate into sexual stages (gametocytes). Each microgametocyte produces approximately 100 motile microgametes, each capable of fertilising a single macrogamete. The ensuing zygote encapsulates itself in a protective wall, becoming an oocyst, and is excreted in the faeces of the definitive host to sporulate in the external environment.

The development of the resilient oocyst wall is a crucial feature of the Coccidia and provides remarkable protection, facilitating its essential transmission between hosts. Moreover, disrupting its formation is the basis for the only subunit vaccine against any apicomplexan disease to reach the marketplace, underscoring the potential for controlling parasitic disease by blocking transmission [5,6]. However, the development of additional transmission blocking strategies is hampered by our limited understanding of the molecular mechanisms that govern gametocyte and oocyst development.

The differentiation and development of distinct biological stages in the Apicomplexa are dependent on regulated gene transcription. Accordingly, profiling quantitative changes in gene transcription has proven a useful strategy for identifying important stage-specific genes in asexual stages of *E. tenella* [7-9] and *T. gondii* [10-13], as well as in gametocyte stages of *Plasmodium* [14,15]. To date, a global analysis of gene transcription has yet to be performed for coccidian gametocytes due, in part, to difficulties producing sufficient quantities of parasite material for conventional transcriptional analysis. However, recent advances in both the sensitivity and affordability of next-generation transcription profiling techniques (i.e. RNA Seq) have opened the door for a thorough analysis of the *E. tenella* gametocyte transcriptome.

Described in the present study are results from an RNA Seq analysis of *E. tenella* gametocytes compared with two asexual stages - sporozoites and merozoites. Many of the upregulated gametocyte transcripts identified encode proteins with known or potential roles in parasite transmission. Two oocyst wall proteins, a subtilisin-like protease and an amine oxidase, all specific to macrogametocytes, are characterised in further biological detail, along with a microgametocyte gamete fusion protein, underscoring their potential as transmission blocking targets.

## Results and discussion

### Transcriptome sequencing of *E. tenella* gametocytes, merozoites and sporozoites

The dimorphic gametocyte stages of *Eimeria* carry out specific biological roles, including fertilisation and oocyst wall assembly, which are required for the formation of an infective (sporulated) oocyst and transmission between hosts. In an effort to better characterise these enigmatic stages, transcriptome sequencing was carried out on *E. tenella* gametocytes, comprising a mixture of both macrogametocytes (female) and microgametocytes (male) (Additional file 1a,b). In parallel, the transcriptomes of two different asexual parasite stages, namely third-generation merozoites (112 h post-infection) and sporozoites (excysted from sporulated oocysts), were also sequenced to allow the identification of upregulated transcripts in gametocytes.

Total RNA was extracted from triplicate biological replicates representing each developmental stage, DNase-treated and quality assessed by automated gel electrophoresis (Additional file 1c). Parasite-specific large ribosomal RNA bands (26S and 18S) were detected in all samples, although trace amounts of contaminating host RNA (28S) were observed in gametocyte samples, an unavoidable feature attributed to the methods used to enrich these stages from infected chickens (see Methods); this was not a strong concern since we performed read mapping to the *E. tenella* genome scaffold and not *de novo* assembly. Paired-end sequencing was carried out on all RNA samples using the Illumina HiSeq platform. Over 10 million paired-end reads were produced per sample with the exception of one merozoite replicate, Mb, which failed outright and was excluded from further analyses (Additional file 1d).

Paired-end reads were mapped to all 8,786 exon models (representing unique proteins) predicted in the *E. tenella* genome scaffold (available on www.toxodb.org). The proportion of reads uniquely mapping to *E. tenella* exon models was lower in gametocyte RNA Seq libraries than in merozoites or sporozoites and can be explained by the higher proportion of contaminating host RNA in the gametocyte samples (Additional file 1c). Nevertheless, the extraordinary depth of sequencing achieved through Illumina HiSeq - including a total of 79.3 million paired-end reads mapped uniquely to *E. tenella* exon models across the three gametocyte libraries – provided a solid foundation for quantitative assessment of gene transcript levels. A table listing the total number of paired reads mapped to each of the 8,786 predicted exon models in each of the nine RNA Seq libraries is provided (Additional file 2).

### Identification of upregulated gametocyte transcripts through analysis of differential expression

The transcription of genes encoding proteins with important roles in *E. tenella* gametocyte biology, including oocyst wall biosynthesis [16], protein glycosylation [17] and proteolytic cleavage of oocyst wall proteins [18], is typically restricted to gametocytes and zygotes (early oocysts). In order to identify other upregulated gametocyte transcripts, RNA Seq mapping was used to generate quantitative differential expression (DE) profiles of individual genes between developmental stages of *E. tenella*.

**Figure 1:**
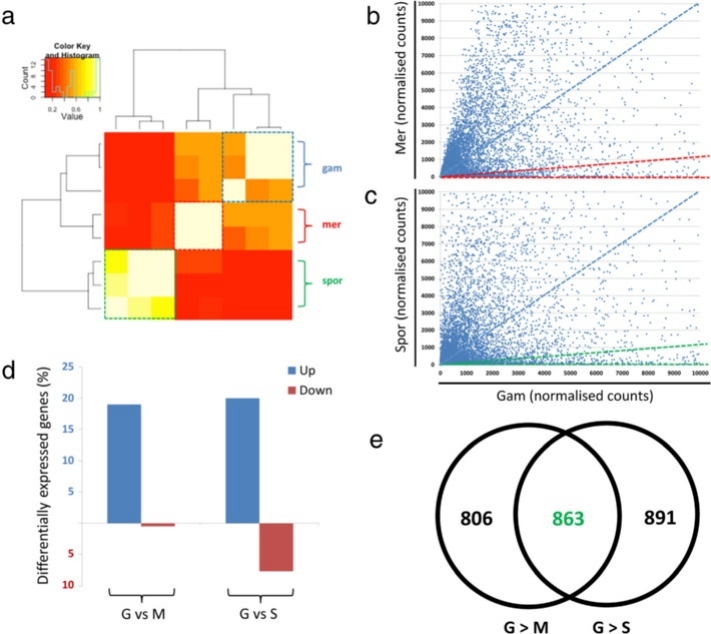
**Identification of upregulated gametocyte transcripts using differential expression analysis. (a)** The eight RNA Seq libraries were subjected to pair-wise hierarchical clustering between the vectors of read counts for all pairs of samples. Heat-mapping of Pearson’s correlation coefficient scores reveal similarities between the transcriptome profiles of the different samples, with white (Pearson R =1.0) being the most similar and red (Pearson R = 0.0) being the least similar. Adjacent dendograms reveal clustering of the biological replicates within the same developmental stages, including gametocytes (gam), merozoites (mer) and sporozoites (spor). (b, c) Normalised read counts of individual genes calculated for gametocytes have been plotted against those for merozoites **(b)** and sporozoites **(c)**. Gene transcripts calculated to be upregulated in gametocytes compared with merozoites or sporozoites reside between dotted red or green dotted-lines, respectively. The dotted blue lines indicate equal levels of transcript abundance between samples. For the sake of clarity, the axis limits have been set to a maximum normalised count of 10,000, inevitably excluding some highly expressed gene transcripts. **(d)** Summary of the DE analysis of gametocytes compared with merozoites (G vs M) or sporozoites (G vs S) showing the percentage of genes upregulated (blue) or downregulated (red) in gametocytes, of all predicted *E. tenella* genes. **(e)** Venn diagram revealing the overlap of genes whose transcript levels were upregulated in gametocytes compared with either merozoites (left, G > M) or sporozoites (right, G > S). A total of 863 upregulated gametocyte transcripts genes were identified in this overlapping region.

However, prior to this DE analysis, the global transcription profiles of each of the eight RNA Seq libraries were compared in a pair-wise manner using hierarchical clustering (described in Methods). Heat-mapping of the Pearson correlation coefficient scores, confirmed that transcription profiles were most similar between replicates of the same biological stage (Figure 1a). The secondary clustering of gametocyte and merozoite replicates is unsurprising given that third-generation merozoites differentiate into gametocytes. Furthermore, this secondary clustering is also caused by the presence of contaminating merozoite stages co-purified in gametocyte samples, an unavoidable bi-product of the asynchronicity of *E. tenella* infections at this later time point (144 h post-infection).

Next, normalised read counts were generated for each of the three different biological stages using the DESeq algorithm [19], thereby allowing a comparison of gene transcript levels between different samples. A table listing the normalised counts calculated for each gene in the three biological stages is provided (Additional file 3). A plot of normalised counts for individual gene transcripts in gametocytes against those in merozoites (Figure 1b) or sporozoites (Figure 1c) gives an overview of global DE of gene transcription. Accordingly, genes transcribed specifically in gametocytes plot along or near the x-axis, or along the y-axis for either merozoites or sporozoites, while genes showing no DE plot along the dotted blue line (*note*, the presence of merozoites in the gametocyte samples results in a shift in the plotting of merozoite-specific genes away from the y-axis). These pairwise comparisons demonstrate that the transcripts of a large population of genes are detected specifically in gametocytes, mirroring the distinct processes utilised in these sexual stages.

For the purposes of defining stage-specific expression, genes were defined as DE if the log2 fold-change calculated from normalised counts between two stages was equal to or greater than three (or a fold-change ≥ eight). The population of upregulated gametocyte transcripts identified using this threshold has been highlighted in the pairwise comparisons with merozoites and sporozoites (dotted red and green lines, respectively – Figure 1b,c). The log2 fold-changes calculated for all genes in DESeq pairwise comparisons between (i) gametocytes and merozoites or (ii) gametocytes and sporozoites are provided (Additional file 3) along with an adjusted p-value representing false discovery rates. Excluding genes with DE false discovery rates of >0.05, 1,669 gene transcripts (19.0% of all predicted *E. tenella* genes) are upregulated in gametocytes compared to merozoites and 1,754 (20.0%) in gametocytes compared to sporozoites (Figure 1d). In contrast, fewer genes were calculated with higher transcript levels in merozoites (44, 0.5%) or sporozoites (675, 7.7%) compared to gametocytes. The identification of 44 merozoite-specific genes is almost certainly a gross underestimate since the co-purification of merozoites in the gametocyte sample (roughly estimated by light microscopy as ~10% of the sample) clearly limits the identification of genes with elevated transcript levels in merozoites. However, in the scope of our study, the identification of genes with elevated transcript levels in gametocytes is highly stringent. In total, 863 upregulated gametocyte transcripts were identified (Figure 1e), representing 9.8% of all predicted *E. tenella* genes. The gene identification, putative biological function and transcript abundance (expressed as FPKM, described below and in the Methods) for each of these genes, in each developmental stage, are provided (Additional file 4).

### Highly abundant upregulated gametocyte transcripts

**Figure 2:**
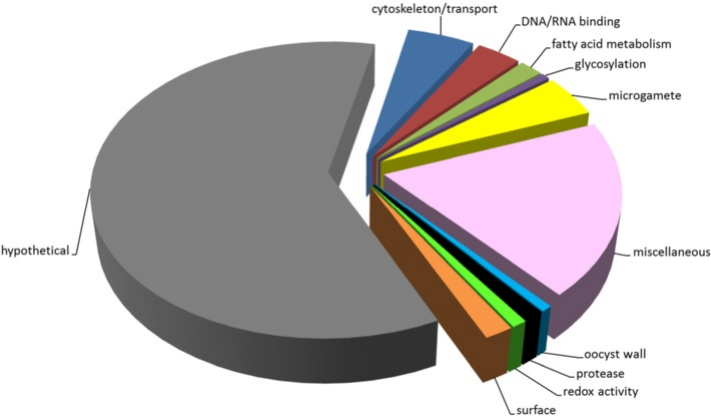
**Biological functions of proteins coded by upregulated gametocyte transcripts.** The biological function of proteins coded by each of the 863 upregulated gametocyte transcripts was assigned manually based on ToxoDB, Blast2Go or annotations published previously (See Additional file 4). The pie chart represents the proportion of upregulated gametocyte transcripts characterised by these different biological functions.

Within the subset of 863 upregulated gametocyte transcripts identified in *E. tenella*, the level of transcript abundance for each gene in gametocytes, expressed as FPKM (Fragments Per Kilobase of exon model per Million mapped reads) varies by many orders of magnitude from 27,715.26 (*ETH_00019840*, hypothetical protein) down to 0.89 (*ETH_00021185*, hypothetical protein) (Additional file 4). A majority (60.4%) of all 863 upregulated gametocyte transcripts code for hypothetical proteins (Figure 2), an unsurprising observation given the limited understanding of coccidian sexual biology. However, genes coding for proteins with putative roles in glycosylation, protease activity, redox activity and fatty acid metabolism and as components of microgametes, surface and the oocyst wall, are highlighted in Figure 2 and are described in further detail below. Other putative functions highlighted but not discussed in detail include: (1) cytoskeleton/transport, processes which may overlap with microgamete flagellar functions; and (2) DNA/RNA binding, which may have unspecified roles in gametocyte-specific gene regulation. The miscellaneous category includes proteins with diverse functions, such as kinase activity, calcium binding, metabolism, etc., whose roles in coccidian gametocyte biology remain unclear and require detailed further study before any meaningful discussion of their functional significance can be attempted.

The two genes with the highest transcript abundance in gametocytes, *ETH_00019840* and *ETH_00019845*, are tandemly located in the *E. tenella* genome (in Supercontig_2) and code for proteins that share almost 100% sequence identity (apart from a 44 amino acid insertion within ETH_00019845) (Additional file 5). Both proteins contain a predicted signal peptide and either four (ETH_00019840) or five (ETH_00019845) transmembrane domains. The third and fourth most abundant upregulated gametocyte transcripts, *ETH_00019835* and *ETH_00032470*, code for proteins with predicted signal peptides and 100% sequence identity over the first 98 amino acids of the N-terminal (Additional file 6). Efforts to characterise the function of these four proteins is on-going.

As expected, genes coding for previously characterised gametocyte antigens and oocyst wall proteins, including EtGAM22, EtGAM56, EtGAM230 and EtGAM59, are among the most highly transcribed gametocyte genes; these are described in greater detail further on. Another highly transcribed gametocyte gene is *ETH_00034390*, which codes for a macrophage migration inhibitory factor, EtMIF, originally described in *E. tenella* merozoites [20]. Interestingly, this protein has been subsequently localised to wall-forming bodies of macrogametes and appears to play a role in modulating the host immune system [21].

### Highly abundant transcripts code for novel macrogamete-specific oocyst wall proteins

**Figure 3:**
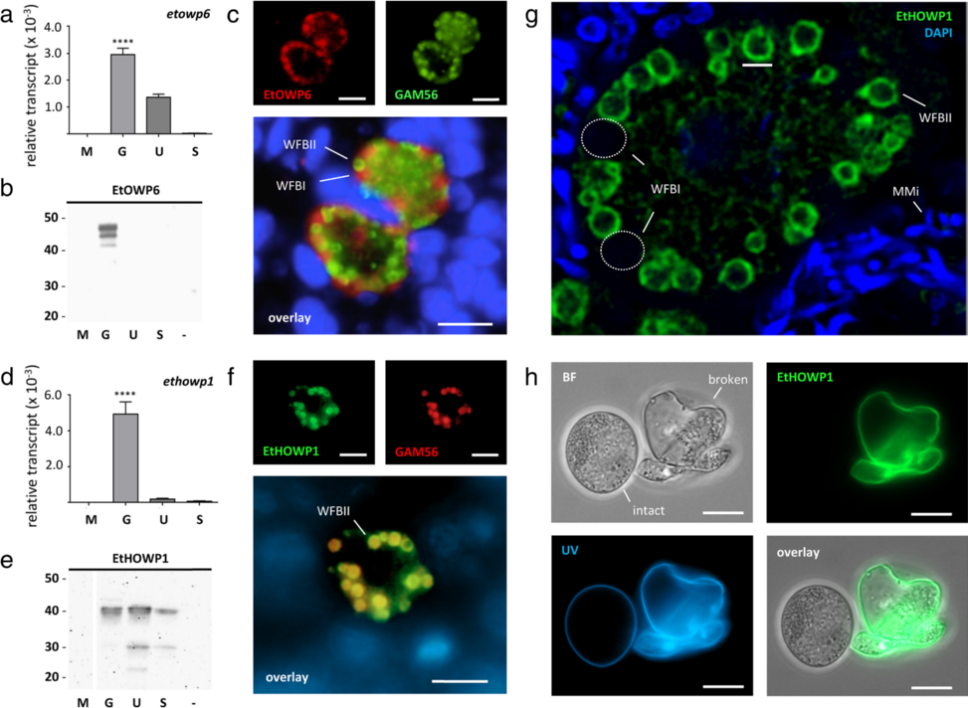
**Differential expression and localisation of** ***E. tenella*** **oocyst wall proteins. (a) (d)** Quantitative reverse-transcriptase PCR was carried out on different developmental stages of *E. tenella*, including merozoites (M), gametocytes (G), unsporulated (U) and sporulated oocysts (S). The relative transcript abundance of *etowp6* **(a)** and *ethowp1* (d) was determined relative to *et18S* small subunit ribosomal RNA for each developmental stage. **** indicates a statistically significant difference of P < 0.001 between one sample and all other samples. **(b) (e)** Different developmental stages of *E. tenella* (M, G, U and S), as well as an uninfected host control (−) were analysed by Western blot using anti-EtOWP6 rabbit sera (diluted 1 in 1,000) **(b)** and anti-EtHOWP1 mouse sera (1 in 4,000) **(e)**. Molecular weight marker is listed to the left in kDa. **(c) (f) (g)** Sections of *E. tenella*-infected chicken caeca (144 h post-infection) were analysed by immunofluorescence microscopy with DAPI counterstaining. **(c)** Anti-EtOWP6 rabbit sera (1 in 200) localises to wall-forming bodies type I (WFBI) while anti-GAM56 mouse sera (1 in 1,000) localises to wall-forming bodies type II (WFBII). Scale bar = 10 μm. **(f)** Anti-EtHOWP1 mouse sera (1 in 2,000) and anti-GAM56 rabbit sera (1 in 1,000) co-localise to WFBII. Scale bar = 10 μm. **(g)** Confocal imaging of anti-EtHOWP1 rabbit sera (1 in 2,000) demonstrates WFBII staining, while WFBI remain unstained. DAPI stains mid-stage microgametes (MMi). Scale bar = 2 μm. **(h)** Broken and intact *E. tenella* unsporulated oocysts were probed with anti-EtHOWP1 (1 in 2,000). Oocyst walls fluoresce blue under UV excitation, due to the presence of cross-linked dityrosine. An overlay of bright field (BF) and anti-EtHOWP1 staining shows localisation to the walls of broken oocysts (the inner layer). Scale bar = 10 μm.

Oocyst wall and surface proteins are attractive transmission blocking targets [22-24]. CoxAbic®, the only commercially-available subunit vaccine against any apicomplexan, comprises macrogamete-specific proteins, GAM56 and GAM82, both of which are destined for incorporation into the oocyst wall [5]. Gene transcripts coding for EtGAM56 and other oocyst wall proteins (EtGAM22, EtGAM59, and EtGAM230) are confirmed here to be upregulated in gametocytes (Table 1). The fifth most abundant upregulated gametocyte transcript detected here, *etgam22*, is a multi-copy gene that codes for a histidine- and proline-rich protein [25], while *etgam56* and *etgam59* code for tyrosine-rich proteins [16,25]. Like GAM56 and GAM82, EtGAM22 is a component of the wall forming bodies of macrogametes and is incorporated into the developing oocyst wall [24,25]. *ETH_00012470* codes for a protein that shows homology to the cysteine-rich oocyst wall proteins, TgOWP6 [26] and CpOWP6 [27] and has been named ‘EtOWP6’. This family of proteins is expressed in wall forming bodies of macrogametes and the oocyst walls of *T. gondii* and *Cryptosporidium parvum* [26,28]. Importantly, EtOWP6 shares the domain architecture described for both TgOWP6 and CpCOWP6 (Additional file 7), specifically the conservation of cysteine-rich Type I repeats [27], supporting its role as a structural component of the oocyst wall. Quantitative RT-PCR confirmed the presence *etowp6* transcript in *E. tenella* gametocytes but also in unsporulated oocysts (Figure 3a). Western blot using anti-EtOWP6 polyclonal antibody detected protein only in gametocyte stages (i.e. not in either oocyst stages), with three bands of 48, 45 and 41 kDa (the predicted size of EtOWP6 is 58.5 kDa) (Figure 3b). It is unclear whether these bands represent different conformations of the one EtOWP6 protein or represent different cross-reactive targets. Immunolocalisation studies of *E. tenella*-infected chicken intestine using this antibody revealed reactivity against wall-forming bodies type I of macrogametes, with GAM56 antibody used as a counterstain for wall-forming bodies type II (WFBII) (Figure 3c). Surprisingly, EtOWP6 could not be detected in oocyst samples by either Western blot (Figure 3b) or immunolocalisation on both broken and unbroken oocysts (data not shown), despite being detected in proteomic analyses of *E. tenella* oocyst walls [29]. One explanation may be that cross-linking of the cysteine-rich protein during oocyst wall assembly drastically reduced the affinity of this particular antibody; although the reduction and alkylation performed prior to Western blot analyses should have been sufficient for breaking disulphide bonds.

**Table 1:** **Upregulated gametocyte transcripts coding for oocyst wall and surface proteins**

**Gene ID**	**FPKM**	**Annotation**	**Function**
ETH_00035480	8555.22	EtGAM22	oocyst wall
ETH_00007320	2961.32	EtGAM56	oocyst wall
ETH_00018895	2836.86	EtHOWP1	oocyst wall
ETH_00012470	1173.37	EtOWP6	oocyst wall
ETH_00016615	844.43	EtGAM230	oocyst wall
ETH_00007315	544.33	EtGAM59	oocyst wall
ETH_00022305	2917.01	surface antigen 7	surface
ETH_00024330	369.46	surface antigen 10	surface
ETH_00026205	311.85	PAN domain-containing protein	surface
ETH_00034935	270.05	surface antigen 10	surface
ETH_00030195	263.42	fasciclin domain-containing protein	surface
ETH_00022280	186.18	surface antigen 7	surface
ETH_00038085	175.61	PAN domain-containing protein	surface
ETH_00012815	172.72	PAN domain-containing protein	surface
ETH_00027460	124.33	PAN domain-containing protein (EC 3.4.21.27), related	surface
ETH_00017025	116.54	plasma-membrane H + −ATPase	surface
ETH_00022855	58.90	plasma membrane calcium-transporting atpase 3-like	surface
ETH_00023630	44.53	cell surface glycoprotein 1	surface
ETH_00016215	17.17	outer membrane protein	surface
ETH_00023605	7.77	PAN domain-containing protein	surface
ETH_00010765	4.13	surface antigen 2	surface

Upregulated gametocyte transcripts coding for known or putative oocyst wall or surface proteins are listed along with their transcript abundance (FPKM), annotation and biological function.

An additional gene, *ETH_00018895*, codes for a protein that was also detected by mass spectrometry in purified oocyst walls of *E. tenella* [29] and is hereto referred to as EtHOWP1, for *E. tenella* Hypothetical Oocyst Wall Protein 1. Unlike *etowp6*, the *ethowp1* transcript was detected exclusively in gametocyte stages of *E. tenella* (Figure 3d). However, Western blot analysis using anti-EtHOWP1 polyclonal antibody revealed a doublet band of approximately 40 kDa in gametocytes, unsporulated oocysts and sporulated oocysts, with additional lower molecular weight bands of 30 kDa and 23 kDa in unsporulated and sporulated oocyst samples (Figure 3e). This banding pattern is reminiscent of the proteolytic processing of tyrosine-rich proteins, GAM56 and GAM82, prior to oocyst wall integration [16]; although, in the case of EtHOWP1, the larger 40 kDa protein band persists in oocyst samples. In contrast to EtHOWP1 protein levels, *ethowp1* transcript was detected only in gametocyte stages, a feature observed previously with *etgam56* [16]. For EtGAM56, the discrepancy between the gene transcript and protein levels is explained by the stockpiling of EtGAM56 protein in WFBII prior to oocyst wall assembly and the high stability of these proteins in the mature oocyst wall structure [16,30]. A similar observation was made for EtHOWP1, which co-localised with EtGAM56 to WFBII (Figure 3f), organelles characterised by their doughnut-like appearance (Figure 3g). WFBII migrate to the periphery of macrogametes and form the inner layer of the developing oocyst wall [31]. Accordingly, when intact and broken oocysts were probed with EtHOWP1 antisera, the protein was detected exclusively in the walls of broken oocysts since the inner layer of the oocyst wall is not accessible in intact oocysts (Figure 3h). In summary, EtHOWP1 is a *bona fide* oocyst wall protein that, looking forward, should be considered as an attractive vaccine candidate.

Also included in Table 1 are genes that code for predicted surface proteins of *E. tenella*. Of particular interest are *ETH_00022305, ETH_00024330, ETH_00034935, ETH_00022280* and *ETH_00010765*, whose cognate proteins share homology with an immunogenic sporozoite surface antigen (SAG) or TA4 [22]. Another subset of predicted surface proteins are those containing PAN domains (ETH_00026205, ETH_00038085, ETH_00012815, ETH_00027460 and ETH_00023605), which are typically found in proteins involved in adhesion, including in microneme proteins (MICs) of *E. tenella* [32]. The role of these putative surface proteins in gametocyte biology remains to be investigated.

### Identification of macrogamete-specific subtilisins and oxidoreductases with putative roles in oocyst wall biosynthesis

**Figure 4:**
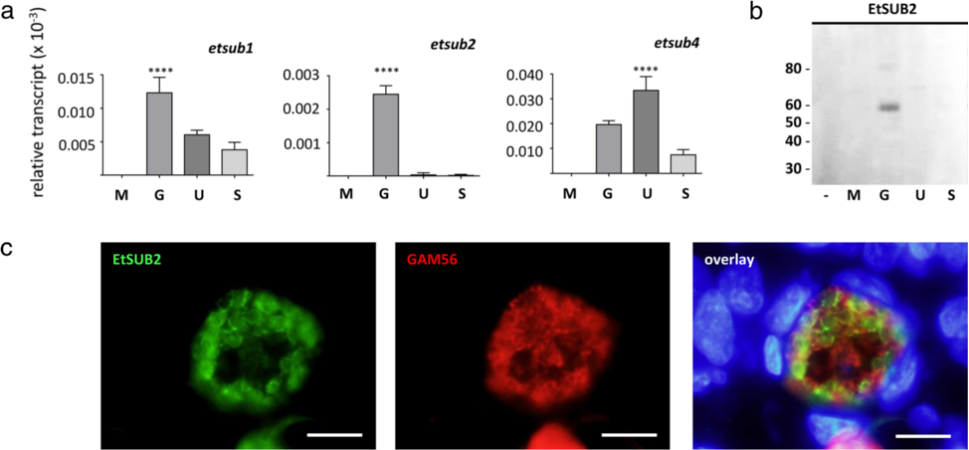
**Differential expression and localisation of** ***E. tenella*** **subtilisin 2 (EtSUB2). (a)** Quantitative reverse-transcriptase PCR was carried out on different developmental stages of *E. tenella*, including merozoites (M), gametocytes (G), unsporulated (U) and sporulated oocysts (S). The relative transcript abundance of *etsub1*, *etsub2* and *etsub4* was determined relative to *et18S* small subunit ribosomal RNA for each developmental stage. **** indicates a statistically significant difference of P < 0.001 between one sample and all other samples. **(b)** Different developmental stages of *E. tenella* (M, G, U and S) and uninfected host (−) were analysed by Western blot using anti-EtSUB2 mouse sera (1 in 500). Molecular weight marker is listed to the left in kDa. **(c)** A section of *E. tenella*-infected chicken caeca (144 h post-infection) was analysed by immunofluorescence microscopy using anti-EtSUB2 mouse sera (1 in 200) and anti-GAM56 rabbit sera (1 in 1,000) with DAPI counterstaining. Scale bar = 5 μm.

While abundant oocyst wall proteins represent the structural building blocks for the coccidian oocyst wall, many of the processes accompanying their incorporation into this bilayered structure are also upregulated during gametocyte development (Table 2). GAM56, for example, undergoes proteolysis into smaller tyrosine-rich peptides, prior to oocyst wall assembly [30]. In the present study, upregulated gametocyte transcripts coding for a number of different proteases were identified including; one serine metalloprotease precursor, one aminopeptidase, an aspartic protease (eimepsin 2) and seven subtilisins. Subtilisins are responsible for processing tyrosine-rich proteins during *E. tenella* gametocyte development [18]. Additional analysis of three subtilisins, *etsub1*, *etsub2* and *etsub4*, demonstrated that *etsub2* is the most specific to gametocytes (Figure 4a); *etsub1* transcript abundance was significantly higher in gametocytes but was still present in oocyst stages, while *etsub4* was significantly higher in unsporulated oocysts than in gametocytes. Western blot analysis using anti-EtSUB2 polyclonal antibody detected a protein of approximately 60 kDa in *E. tenella* gametocytes only (Figure 4b); the predicted size of 48.3 kDa is probably an underestimate due to the truncated gene model of ETH_00025145. Furthermore, immunolocalisation studies using the same antibody revealed localisation of this serine protease to *E. tenella* macrogametocytes (Figure 4c), specifically to the periphery of the parasite cell and adjacent to WFBII (labelled with anti-GAM56).

**Table 2:** **Upregulated gametocyte transcripts coding for proteins involved in the biosynthesis of the oocyst wall**

**Gene ID**	**FPKM**	**Annotation**	**Function**
ETH_00005950	651.50	serine metalloprotease precursor	protease
ETH_00013105	319.88	aminopeptidase N 1	protease
ETH_00007420	113.15	Eimepsin 2 (aspartic protease)	protease
ETH_00000180	76.59	subtilisin-like serine protease	protease
ETH_00006825	69.68	subtilisin 4	protease
ETH_00009270	59.38	subtilisin-like serine protease	protease
ETH_00000395	59.33	subtilisin-like serine protease	protease
ETH_00042675	41.92	subtilisin sub8	protease
ETH_00025145	39.13	subtilisin 2	protease
ETH_00009790	31.77	subtilisin 1	protease
ETH_00034420	722.78	thioredoxin	oxidoreductase
ETH_00033360	475.71	gmc oxidoreductase	oxidoreductase
ETH_00032305	438.54	peroxidoxin 2	oxidoreductase
ETH_00007785	343.29	alkyldihydroxyacetonephosphate synthase, peroxisomal	oxidoreductase
ETH_00025705	332.68	thioredoxin	oxidoreductase
ETH_00028385	311.75	amiloride-sensitive amine oxidase, copper-containing	oxidoreductase
ETH_00027865	282.95	peroxiredoxin 3	oxidoreductase
ETH_00027760	79.18	mannitol 2-dehydrogenase	oxidoreductase
ETH_00015660	153.33	UDP-glucose 4-epimerase	glycosylation
ETH_00007235	58.24	oligosaccharyl transferase STT3	glycosylation
ETH_00019125	52.30	glucosamine-fructose-6-phosphate aminotransferase	glycosylation
ETH_00005370	52.07	UDP-N-acetyl-D-galactosamine:polypeptide N-acetylgalactosaminyltransferase T3	glycosylation
ETH_00007230	44.54	dolichyl-diphosphooligosaccharide--protein glycosyltransferase subunit stt3a	glycosylation
ETH_00018780	44.49	beta-n-acetylglucosaminyltransferase-like protein	glycosylation
ETH_00004905	653.67	GNS1/SUR4 family	fatty acid metabolism
ETH_00007660	497.09	acyl-CoA-binding protein	fatty acid metabolism
ETH_00032045	251.30	acyl-coenzyme A oxidase	fatty acid metabolism
ETH_00035050	179.62	diacylglycerol acyltransferase	fatty acid metabolism
ETH_00019415	135.48	lipase class 3	fatty acid metabolism
ETH_00019675	113.45	very long-chain acyl-CoA synthetase	fatty acid metabolism
ETH_00029525	103.42	acyl-CoA-binding protein	fatty acid metabolism
ETH_00011255	101.30	acyl-protein thioesterase 1-like	fatty acid metabolism
ETH_00025420	62.41	peroxisomal multifunctional enzyme	fatty acid metabolism
ETH_00015480	53.04	EtPKS1 (polyketide synthase)	fatty acid metabolism
ETH_00015230	23.12	peroxisomal 2,4-dienoyl CoA reductase	fatty acid metabolism
ETH_00005790	12.53	EtPKS2 (polyketide synthase)	fatty acid metabolism
ETH_00015485	11.34	EtPKS1 (polyketide synthase)	fatty acid metabolism
ETH_00005575	8.44	enoyl-CoA hydratase/isomerase family protein	fatty acid metabolism
ETH_00043845	8.02	polyketide synthase	fatty acid metabolism
ETH_00005785	3.72	EtPKS2 (polyketide synthase)	fatty acid metabolism

Upregulated gametocyte transcripts coding for proteins with either a known or putative role in oocyst wall biosynthesis are listed along with their transcript abundance (FPKM), annotation and biological function.

**Figure 5:**
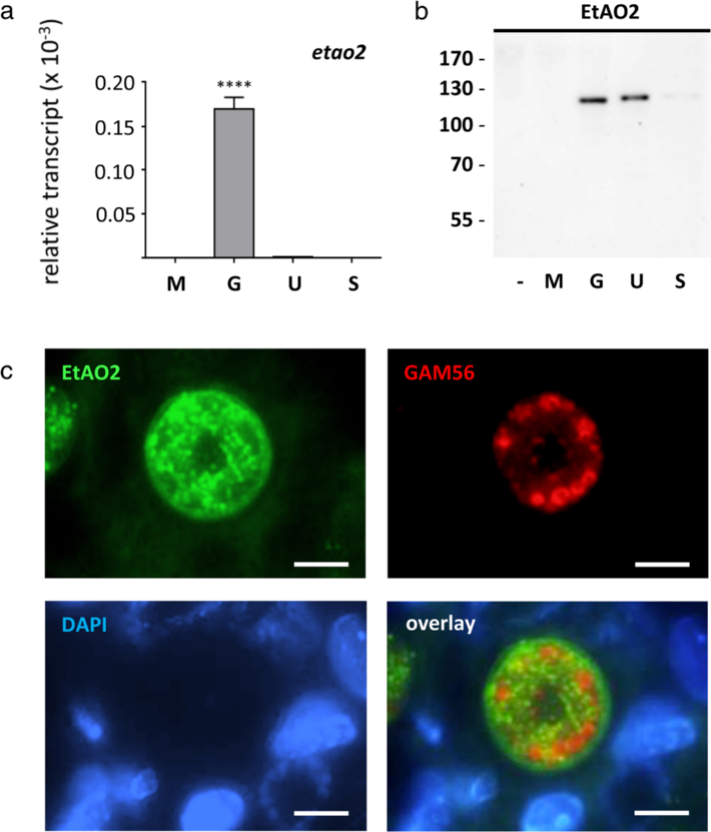
**Differential expression and localisation of** ***E. tenella*** **amine oxidase 2 (EtAO2). (a)** Quantitative reverse-transcriptase PCR was carried out on different developmental stages of *E. tenella*, including merozoites (M), gametocytes (G), unsporulated (U) and sporulated oocysts (S). The relative transcript abundance of *etao2* was determined relative to *et18S* small subunit ribosomal RNA for each developmental stage. **** indicates a statistically significant difference of P < 0.001 between one sample and all other samples. **(b)** Different developmental stages of *E. tenella* (M, G, U and S) and uninfected host (−) were analysed by Western blot using anti-EtAO2 mouse sera (1 in 1,000). Molecular weight marker is listed to the left in kDa. **(c)** A section of *E. tenella*-infected chicken caeca (144 h post-infection) was analysed by immunofluorescence microscopy using anti-EtAO2 mouse sera (1 in 500) and anti-GAM56 rabbit sera (1 in 1,000) with DAPI counterstaining. Scale bar = 5 μm.

Cross-linking of the smaller tyrosine-rich proteins into dityrosine imparts further stability to the oocyst wall and evidence implicates the role of peroxidases in catalysing this reaction [33]. Table 2 includes two peroxiredoxins (peroxidases), in addition to six genes coding for proteins with predicted oxidoreductase activities, potentially capable of cross-linking dityrosine. The amiloride-sensitive amine oxidase, copper-containing protein (EtAO2, coded by *ETH_00028385*) is of particular interest since its possession of an extracellular MAM (meprin, A5, μ)-domain [34] could place it at the site of oocyst wall biogenesis. Quantitative RT-PCR confirmed *etao2* as an upregulated gametocyte transcript (Figure 5a). However, Western blot analysis using anti-EtAO2 polyclonal antibody detected a single 120 kDa protein in mature gametocytes and unsporulated oocysts (Figure 5b). Immunolocalisation studies with this antibody reveal that EtAO2 localises to macrogametes (Figure 5c). Although EtAO2 appears to localise as foci within the cytoplasm, the staining pattern is distinct from the WFBI or WFBII. Aside from some augmented staining at the periphery of mature macrogametes (Figure 5c), there was no observable staining to oocyst stages in tissue sections as might be expected of for an enzyme involved in oocyst wall assembly. It should be noted, however, that given the expected size of EtAO2 is predicted at 183 kDa (~60 kDa larger than observed by Western blot), some caution must be taken in interpreting results obtained with this antibody.

GAM56 and GAM82 have both been shown to be heavily glycosylated [35], a process that is linked to a co-regulated glycosylation pathway in *Eimeria* [17]. In the present study, six upregulated gametocyte transcripts were identified that code for enzymes in the protein glycosylation pathway, including EtGFAT (glucosamine: fructose-6-phophate aminotransferase) (ETH_00019125), the primary enzyme in the amino sugar biosynthesis pathway that was previously localised to *E. tenella* macrogametes [17]. Interestingly, recent studies have indicated the coccidian oocyst wall architecture is comprised not only of glycoproteins but also interlayered β-1,3-glucan fibrils [36] and an outer layer of acid-fast lipids [37]. Glucan synthase, the enzyme catalysing the synthesis of β-1,3-glucan fibrils, is coded by the gene *ETH_00000330* whose transcript levels were upregulated 14-fold in *E. tenella* gametocytes compared to merozoites (note, *ETH_00000330* was not strictly listed as an ‘upregulated gametocyte transcript’ since gametocyte transcript abundance was only four-fold higher than that of sporozoites). A total of sixteen upregulated gametocyte transcripts code for proteins with predicted roles in synthesising or re-modelling the acid-fast lipid layer of the *E. tenella* oocyst wall [37], including the previously described polyketide synthases, EtPKS1 (predicted from both *ETH_00015480* and *ETH_00015485*) and EtPKS2 (*ETH_00005790* and *ETH_00005785*). Note, the upregulated gametocyte transcript *ETH_00043845* appears to code for an additional, as yet undescribed PKS.

Targeted inhibition of highly-divergent, parasite-specific biochemical pathways is yet another strategy for combating coccidian transmission; inhibition of glucan synthase has shown promise in reducing oocyst output in *E. tenella* [36]. The potential to block oocyst wall formation by inhibition of gametocyte-specific subtilisins and oxidoreductases warrants further investigation.

### Microgamete biology is underscored by numerous gametocyte targets, including the gamete fusion protein EtHAP2, which localises to *E. tenella* microgametes

Structural studies of *E. tenella* microgametes describe terminally differentiated stages comprised of a nucleus, a mitochondrion, a number of longitudinally running microtubules and two flagella (reviewed in [4]); in many respects their biology is analogous to that of eukaryotic sperm. While characterisation of microgamete-specific markers in the Coccidia is limited, proteomic analyses of purified microgametes in the related apicomplexan, *Plasmodium berghei*, has highlighted an important role for proteins involved in DNA replication and the axoneme, a specialised cytoskeletal structure of eukaryotic flagella [38,39]. In the present study, genes coding for highly-conserved axoneme-associated proteins, including twenty dyneins, two radial spoke head proteins, two basal body proteins and one central apparatus protein were identified as upregulated gametocyte transcripts (Table 3). *ETH_00013995*, the most abundant of these microgamete-specific transcripts, codes for an armadillo/beta-catenin-like repeat protein that is a homologue of PF16, a microgamete protein of *Plasmodium falciparum*. PF16 is a component of the axoneme central apparatus and is essential for microgamete motility and fertilisation [40]. Likewise, ETH_00022085 shows homology to a protein, TAX-2 (Trypanosome Axonemal Protein-2), required for motility in the flagellated parasite, *Trypanosoma brucei* [41]. Also listed in Table 3 are genes coding for an additional eight flagellar-associated proteins and for proteins with predicted non-flagellar functions. Protamine (ETH_00009265), for instance, is a histone-like protein that binds sperm DNA, condensing the genome into an inactive state [42], while calmegin (ETH_00022665) is a testis-specific ER protein that functions as a protein chaperone and is required for fertility [43,44]. AAT-1 (AMY-1-associated protein expressed in testes-1) protein (ETH_00005660) is predicted to have versatile functions in spermatogenesis, including the modulation of energy required for fertilisation [45], while enkurin (ETH_00003635) is expressed in mammalian sperm and is thought to be involved in signal transduction pathways of fertilisation during gamete fusion [46].

**Table 3:** **Upregulated gametocyte transcripts coding for microgamete proteins**

**Gene ID**	**FPKM**	**Annotation**	**Function**
ETH_00013995	223.15	armadillo/beta-catenin-like repeat protein	axoneme
ETH_00014050	195.46	radial spoke head	axoneme
ETH_00014055	184.22	radial spoke head protein 9 homolog	axoneme
ETH_00025255	125.32	dynein intermediate chain, putative	axoneme
ETH_00008420	99.32	flagellar outer dynein arm light chain 2, putative	axoneme
ETH_00002085	81.54	ciliary basal body-associated, B9 protein	axoneme
ETH_00023565	60.23	flagellar inner arm dynein 1 heavy chain beta	axoneme
ETH_00008820	50.32	axonemal dynein gamma heavy	axoneme
ETH_00008815	45.22	axonemal dynein gamma heavy	axoneme
ETH_00017815	30.16	dynein heavy	axoneme
ETH_00008810	29.97	axonemal dynein gamma heavy	axoneme
ETH_00022085	28.85	cytochrome b5 domain-containing protein 1-like	axoneme
ETH_00027240	23.02	flagellar inner arm dynein 1 heavy chain alpha	axoneme
ETH_00023560	22.57	dynein heavy chain axonemal	axoneme
ETH_00022700	20.51	dynein heavy chain axonemal	axoneme
ETH_00008805	20.26	dynein heavy chain family protein	axoneme
ETH_00023555	18.16	dynein heavy	axoneme
ETH_00043205	16.08	dynein heavy chain axonemal-like	axoneme
ETH_00008800	14.75	dynein heavy chain	axoneme
ETH_00023545	14.07	flagellar inner arm dynein 1 heavy chain beta	axoneme
ETH_00008795	13.24	flagellar outer dynein arm heavy chain gamma	axoneme
ETH_00027230	12.44	dynein heavy chain	axoneme
ETH_00017810	11.97	dynein heavy chain family protein	axoneme
ETH_00039340	11.06	flagellar basal body protein	axoneme
ETH_00027220	9.11	flagellar inner arm dynein 1 heavy chain alpha	axoneme
ETH_00027225	7.60	flagellar inner arm dynein 1 heavy chain alpha	axoneme
ETH_00030810	115.85	flagellar associated protein	flagella
ETH_00023960	55.80	flagellar associated protein	flagella
ETH_00013980	53.78	flagellar associated protein	flagella
ETH_00023965	50.49	flagellar associated protein	flagella
ETH_00019365	42.87	flagellar associated related	flagella
ETH_00004845	41.61	flagellar associated protein	flagella
ETH_00019370	31.03	flagellar associated related	flagella
ETH_00002895	22.43	testis-expressed sequence 9 protein	flagella
ETH_00009265	53.51	protamine P1 protein, putative	DNA condensation
ETH_00003635	232.25	enkurin-related protein	gamete fusion
ETH_00017050	107.31	generative cell specific-1 (HAP2)	gamete fusion
ETH_00017055	102.53	generative cell specific-1 (HAP2)	gamete fusion
ETH_00022665	71.08	calmegin, putative	protein chaperone
ETH_00005660	64.65	AMY-1-associated protein expressed in testes	energy

Upregulated gametocyte transcripts coding for proteins with a putative role in microgamete biology are listed along with their transcript abundance (FPKM), annotation and biological function.

**Figure 6:**
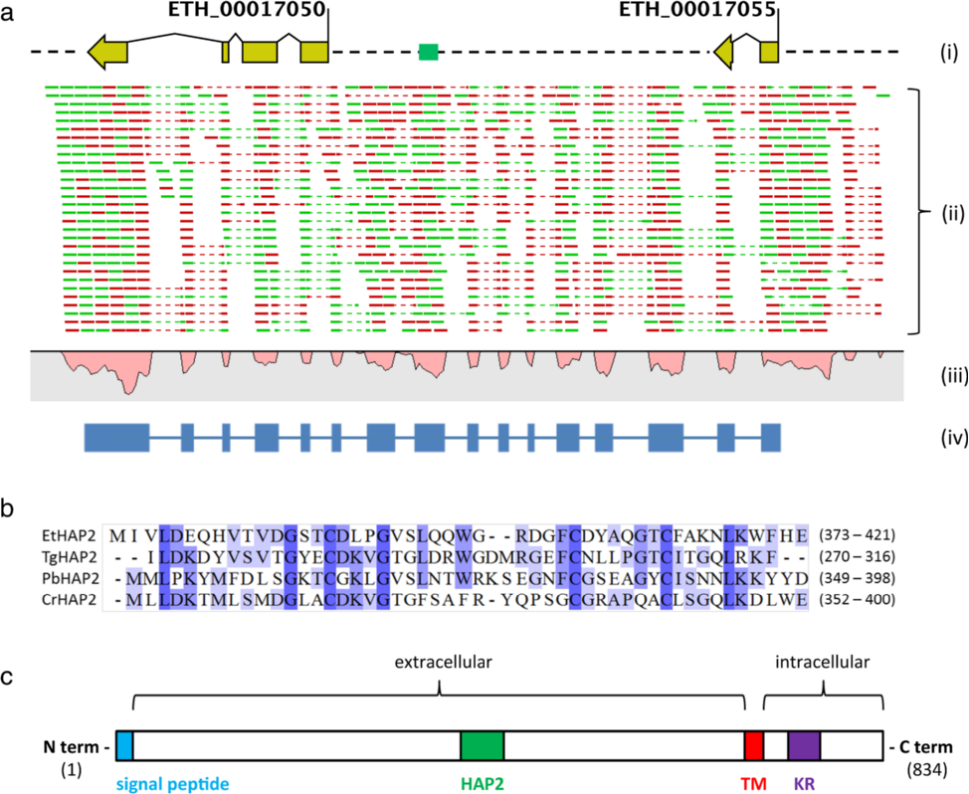
**New gene model codes for a canonical GCS1-HAP2 protein, EtHAP2. (a)** (i) Upregulated gametocyte transcripts, *ETH_00017050* and *ETH_00017055*, were originally predicted as separate, tandemly positioned genes. Exons (yellow) are separated by introns (solid black line), while the intergenic regions are indicated by a broken black line. The position of the genomic sequence coding for a putative GCS1-HAP2 domain is indicated by a green box. (ii) Gametocyte RNA Seq reads were mapped to the *E. tenella* genome in either a 5’ – 3’ (green lines) or 3’ – 5’ (red lines) orientation. Gaps in the mapping of individual reads (dotted lines) indicate the putative position of introns. (iii) Coverage of mapped reads (y-axis, in reverse) also indicates the position of putative exons. (iv) RT-PCR revealed that *ETH_00017050* and *ETH_00017055* are actually part of a single *ethap2* transcript. Exons of this transcript are indicated as blue boxes, while separating introns are indicated as a blue line. The resulting EtHAP2 coding sequence reads from right (5’) to left (3’). **(b)** Multiple alignment of amino acid sequences corresponding to the HAP2 domains of *E. tenella* (EtHAP2), *T. gondii* (TGME49_285940, TgHAP2), *P. berghei* (PBANKA_121260, PbHAP2) and *C. reinhardtii* (C_530033, CrHAP2). Blue shading is proportional to levels of conservation between the four species. Amino acid sequence limits are indicated to the right. **(c)** The domain architecture of the EtHAP2 protein is illustrated (to scale). An N-terminal signal peptide is followed by an extracellular region (comprising the HAP2 domain), a transmembrane domain (TM), and a C-terminal intracellular domain containing a lysine/arginine-rich domain (KR).

Finally, two upregulated gametocyte transcripts, *ETH_00017050* and *ETH_00017055*, are tandemly located in the *E. tenella* genome and code for proteins with homology to a family of transmembrane proteins, known as HAP2-GCS1 (HAPLESS2/Generative Cell Specific) whose expression is restricted to male gametes in a range of different organisms [47]. Each of these genes aligns to different regions of an *E. tenella* ‘GCS1’ mRNA sequence (GenBank: AB723702.1, direct submission, unpublished) implying a misprediction of both *ETH_00017050* and *ETH_00017055* exon models (data not shown). Mapping of gametocyte RNA Seq reads to the *E. tenella* genome supported that the two HAP2-like genes are actually part of one contiguous gene transcript (Figure 6a); in fact, the canonical HAP2 domain is located in what is currently predicted as an intergenic region of the *E. tenella* genome, highlighting the current pitfalls in gene prediction models not informed by transcriptome data. Subsequent RT-PCR and sequencing of the *ethap2* transcript confirmed this observation and revealed 100% of identity to the GenBank mRNA sequence, herein used as the EtHAP2 CDS.

The expression of HAP2 in *Plasmodium* is restricted to microgametes and is required for gamete fusion and subsequent fertilisation [48-50]. The HAP2 domains of EtHAP2 and PbHAP2 are partially conserved (33% sequence identity), with further sequence identity being observed in the HAP2 domains of CrHAP2 (from the green alga *Chlamydomonas reinhardtii*; 27%) [48] and a putative TgHAP2 (*T. gondii*; 42%) (Figure 6b). Although sequence homology outside this domain is limited, the general architecture of the EtHAP2 protein, including the presence of a signal peptide, extracellular HAP2 domain, single transmembrane domain and an intracellular, positively-charged (lysine/arginine-rich) C-terminal region (Figure 6c), is as described previously for HAP2-GSC1 proteins [51].

**Figure 7:**
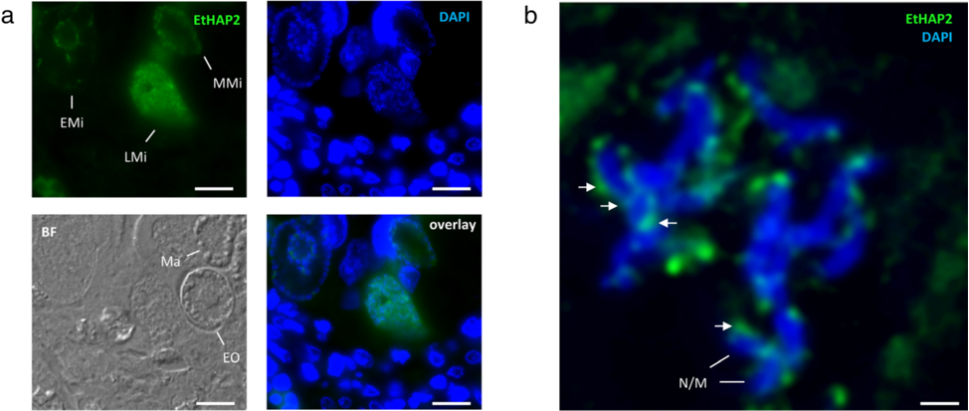
**EtHAP2 expression is restricted to** ***E. tenella*** **microgametes. (a)** Sections of *E. tenella*-infected chicken caeca (144 h post-infection) were stained with anti-EtHAP2 mouse sera (1 in 100) and DAPI. EtHAP2 (green) localised with increasing intensity to DAPI-positive early-, mid- and late-stage microgametocytes (EMi, MMi and LMi, respectively). Macrogametes (Ma) and early oocysts (EO), visible under bright field (BF), were EtHAP2-negative. Scale bar = 10 μm. **(b)** The same tissue section was imaged using confocal microscopy and post-acquisition deconvolution. The nucleus and mitochondrion (N/M) of individual late-stage microgametes stain DAPI-positive and appear continuous and elongated. EtHAP2 (green) localisation (arrows) is distinct but often adjacent to N/M, consistent with either cytosolic or surface localisation. Scale bar = 1 μm.

The extracellular HAP2 domain is thought to act as a fusogen, mediating membrane fusion between mating gametes [47]. Antibodies raised against the HAP2 domain of EtHAP2 reacted specifically with microgametes of *E. tenella*, with no observable reactivity with either macrogametes or developing oocysts (Figure 7a). EtHAP2 staining appeared more intense in late-stage (mature) microgametes than either mid-stage or early-stage microgametes, consistent with a predicted role in fertilisation. Efforts to determine the subcellular localisation of EtHAP2 revealed a distribution pattern distinct from that of the microgamete nucleus and mitochondrion, organelles that appear as near-continuous, DAPI-positive structures (Figure 7b). Beyond this, it was difficult to confirm whether EtHAP2 localisation was cytoplasmic, surface or, less likely, as part of the flagella. In the flowering plant, *Arabidopsis thaliana*, HAP2 redistributes from the cytoplasm to the surface of sperm cells upon contact with a cysteine-rich protein and potential ligand, EC-1 (Egg Cell 1) [52]. Likewise, it is conceivable that EtHAP2 is exposed at the surface of microgametes only after attachment to macrogametes during fertilisation.

Evidence suggests that unfertilised *Eimeria* macrogametes fail to develop into mature oocysts and/or are non-infective due to a failure to sporulate [53,54]. Therefore, disturbing the biological processes of microgametes, including motility and gamete fusion, is another promising strategy to block the transmission of apicomplexan parasites.

### HOWP1 and AO2 are macrogametocyte proteins that are conserved in *T. gondii*

**Figure 8:**
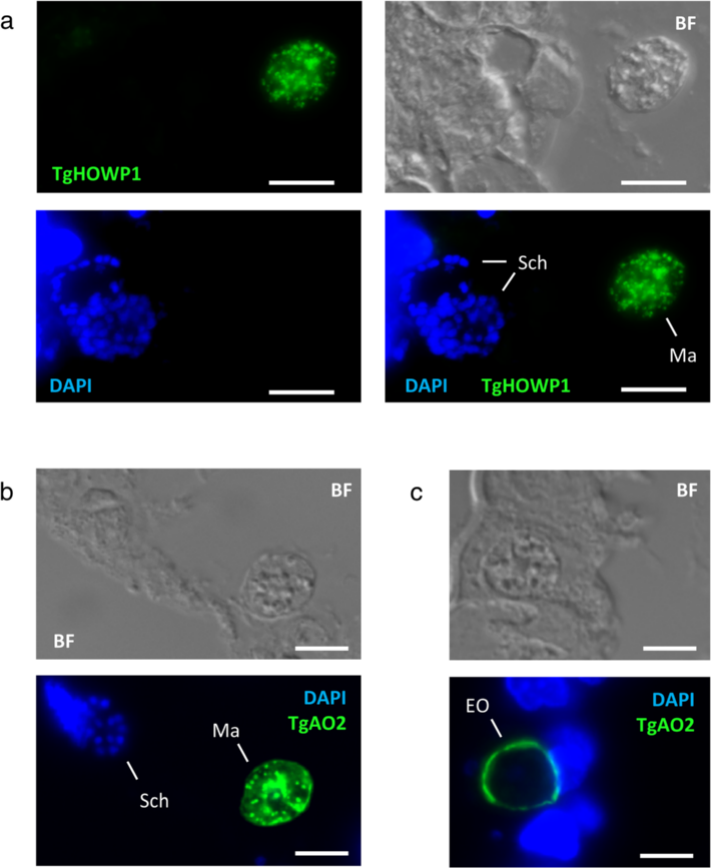
**TgHOWP1 and TgAO2 localisation in** ***T. gondii*** **-infected cat intestine.** Sections of *T. gondii*-infected cat intestine (day 7 post-infection) were analysed by immunofluorescence microscopy with DAPI counterstaining. **(a)** Anti-TgHOWP1 mouse sera (1 in 100) localised to *T. gondii* macrogametes (Ma) but not to DAPI-positive late schizonts (Sch). Scale bar = 5 μm. **(b)** Anti-TgAO2 (1 in 100) localised to *T. gondii* macrogametes (Ma) but not to DAPI-positive late schizonts (Sch). Scale bar = 5 μm. **(c)** Anti-TgAO2 also localised to early oocysts (EO). Scale bar = 5 μm.

The fundamental biological processes of sexual stage development and oocyst formation are broadly conserved in the Coccidia. It would be expected, therefore, that many of the *E. tenella* gametocyte proteins identified in the present study would be biologically conserved in *T. gondii*. Indeed, sequence homology searching of the *T. gondii* genome database (www.toxodb.org) revealed that TGME49_316890 (referred to herein as TgHOWP1) may be the structural orthologue of EtHOWP1, although the two proteins share only 24.7% sequence identity (and a predicted signal peptide) (Additional file 8). Importantly, TgHOWP1 was identified previously in a proteomic analysis of purified *T. gondii* oocyst walls [55]. In the present study, polyclonal antisera raised against a TgHOWP1-GST fusion protein specifically stained *T. gondii* macrogametes in sections of infected cat intestine, while merozoites (contained within late schizonts) remained unstained (Figure 8a). TgHOWP1 appeared to localise to organelles resembling wall forming bodies, although the lack of a WFB marker in *T. gondii* makes this observation somewhat presumptive.

A putative orthologue of EtAO2 was also identified in *T. gondii* (TgAO2), although like EtHAP2, the protein is predicted to be encoded by two adjacent genes, *TGME49_286782* and *TGME49_286778*. Preliminary data from RNA Seq analysis of *T. gondii* gametocytes (unpublished observations) confirms that these two exon models actually represent a single *tgao2* gene transcript, as predicted in a previous version of the *T. gondii* genome assembly (*TGME49_ 086780*). The protein sequences of EtAO2 and TgAO2 (using the TGME49_086780 model) share 59.7% sequence identity, although this extends to 69.8% for the extracellular MAM domain and 70.5% for the copper amine oxidase domain (Additional file 9). Antibody raised against a TgAO2-GST fusion protein corresponding to the entire MAM domain reacted specifically against *T. gondii* macrogametes, like EtAO2, localising to distinct cytoplasmic foci (Figure 8b). In contrast to EtAO2, additional staining was also observed on the surface of early oocysts (Figure 8c), consistent with a possible role for TgAO2 in cross-linking tyrosine-rich proteins in the developing oocyst wall of *T. gondii*.

## Conclusions

In this study, a comparative RNA Seq transcriptomics approach led to the identification of hundreds of genes specifically expressed in *E. tenella* gametocytes. This data set represents a snapshot of the mechanisms at play in coccidian sexual biology and also highlights previously undescribed transmission blocking targets – in particular, two oocyst wall proteins, a subtilisin, an oxidoreductase and a microgamete-specific gamete fusion protein. With so many hypothetical genes identified, further functional characterisation will be paramount for determining the key players in distinct sexual stage processes, including microgamete motility, fertilization and oocyst wall biogenesis. As the transmission blocking potential of these *Eimeria* targets are assessed in future studies, wider implications can be expected in related Coccidia, like *Toxoplasma*, where transmission blocking remains a relatively unexplored control strategy.

## Methods

### Parasites

One day-old chicks (Australorp; Barter and Sons Hatchery, Luddenham, Australia) were housed at the Ernst Facility Animal House (University of Technology, Sydney), under heat lamps for 2 weeks and at 21°C thereafter, with a 12-hour light/dark cycle with free access to food and water. To confirm chickens as free of infection prior to experimental inoculation, faeces were analysed by salt flotation and light microscopy to ensure the absence of oocysts [56]. Chickens were infected orally at 4 to 5 week old chickens were used of age with 2.5 × 10^3^ sporulated oocysts of *Eimeria tenella* (Houghton strain, originally provided by Janene Bumstead, Institute for Animal Health, Compton, UK). Fresh *E. tenella* oocysts were harvested 7 days post-infection (p.i.) from the caeca following protocols described previously [57]. Oocyst sporulation was carried out at 28°C for 72–120 h using a low-pressure aquarium pump for aeration. Sporulated oocysts were then treated with 2.8 M NaCl and 2% sodium hypochlorite (Milton solution) and stored in 2% potassium dichromate at 4°C. Unsporulated oocysts were also treated with Milton solution and stored at −80°C. Merozoites (112 h p.i.) and gametocytes (134 h and 144 h p.i.) were isolated from infected chicken caeca following techniques published previously [23,56]. Sporozoites were excysted from purified sporulated oocysts as described previously [58]. Whole caecal samples were taken at 144 h p.i. for immunolocalisation studies. The samples were stored in 2% paraformaldehyde/0.1 M phosphate buffer (pH 7.4) and embedded in paraffin.

Paraffin blocks of cat intestine infected with the type II CZ *T. gondii* isolate, originally isolated from the faeces of a captive Siberian tiger (*Panthera tigris altaica*) at the Dvůr Králové Zoo (Czech Republic); parasites were passed once through mice to produce bradyzoite-containing tissue cysts and once through cats to generate ~50 million CZ oocysts, which were used to infect four sheep. Brain tissue from sheep containing bradyzoites in tissue cysts was fed to cats. At day 7 post-infection, the small intestine was removed, stored in 4% formaldehyde/0.1 M phosphate buffer (pH 7.4) and embedded in paraffin for future immunolocalisation experiments.

### Total RNA preparation

Parasite samples were resuspended in Trizol® Reagent (Invitrogen) for total RNA extraction. Merozoite, gametocyte and excysted sporozoite samples were homogenised by repeated pipetting while unsporulated and sporulated oocysts were homogenised by vortexing with an equal volume glass beads (710–1,180 μm, Sigma) until oocyst and sporocyst walls were completely broken (no more than ten cycles of 1 min on, 1 min off). Total RNA was purified from homogenised samples as per manufacturer’s instructions. Quantification of total RNA was carried out using a Qubit fluorometer (Invitrogen) Assessment of total RNA quality was carried out on a Bioanalyzer 2100 (Agilent) using an Agilent RNA 6000 Pico Kit. Prior to either RNA Seq or cDNA synthesis, total RNA was treated with RNase-free DNase I (Qiagen) on an RNeasy column (Qiagen), re-purified and, in the case of RNA Seq, re-assessed on a Bioanalyzer 2100.

### cDNA library construction and RNA Seq

Genome-wide transcriptome libraries were produced from triplicate biological replicates of *E. tenella* merozoites, gametocytes and sporozoites. Approximately 200 ng of mRNA were enriched from 15–20 μg of total RNA using the TruSeq RNA Sample Preparation Kit (Illumina). cDNA synthesis and amplification for unstranded, paired-end sequencing was carried out using the Encore® Complete RNA-Seq Library Systems kit (NuGEN). cDNA libraries were selected for fragment sizes of between 150–250 bp and amplified for 15–18 cycles according to the Encore® protocol. Library size and concentration were assessed using a Bioanalyzer 2100 (Agilent) and a Qubit fluorometer (Invitrogen), respectively. Transcriptome libraries were sequenced using the HiSeq 2000 Sequencing System (Illumina). Each library was run on two lanes in a flow cell in order to maximise the total number of RNA Seq reads. The combined RNA Seq run resulted in nine separate fastq files representing each library, containing sequenced paired reads and quality control data.

For mapping, individual reads were aligned to the *E. tenella* Houghton strain genome version 8.1 (available on www.toxodb.org) of the recently published *E. tenella* (Houghton strain) genome sequence [59] using TopHat2 [60]. The counts of reads mapped to gene models were processed by Bioconductor library DESeq [19], producing a table that includes the total mapped reads per gene model for each library, in addition to the normalised read count per gene model for each biological stage (averaged from biological replicates). Fold changes, log2 fold-changes, p-values and adjusted p-values (adjusted for multiple testing with the Benjamini-Hochberg procedure which controls for false discovery rate [61]) are also reported. In the present study, a fold change of equal to or greater than eight (log2 fold change ≥ 3) was used to identify differentially expressed genes, excluding false positives represented by a p-adjusted value cut-off of 0.05 or over. Pair-wise hierarchical clustering between the vectors of read counts of all pairs of samples was carried out using the heatmap.2 function (available in the gplots package from CRAN, cran.r-project.org) according to default settings. An additional measurement of transcript abundance, FPKM (fragments per kilobase of exon model per million mapped reads), was calculated for each gene using the formula “FPKM = 10^6^ × C/(N × L)” where C is the number of paired-reads mapped to the exon model of a gene, N is the total number of mapped reads in each library and L is the length of the gene in kilobases.

### Quantitative reverse-transcriptase PCR (qRT-PCR)

The cDNA samples were synthesised from DNase-treated total RNA using the Superscript^TM^ III First-Strand Synthesis System (Invitrogen), using random hexamer primers. Quantitative RT-PCR was carried out on a Rotor-Gene Q real-time PCR system (Qiagen). Individual reactions were prepared with 0.5 μM of each primer, 5 ng of cDNA and SYBR Green PCR master mix (Qiagen) to a final volume of 20 μl. All experiments were performed twice with separate biological replicates. For each experiment, reactions were performed in triplicate and expression of individual genes was normalised to Ct values of *E. tenella* small ribosomal subunit *18S* RNA using the delta Ct formula: 2^-(Ct*gene* – Ct*et18S*)^. All primers were designed using Primer3 v. 0.4.0 (http://bioinfo.ut.ee/primer3-0.4.0/) and are listed in Additional file 10. Amplified products were sub-cloned into pGEM®-T Easy vector (Promega) as per manufacturer’s instructions and sequenced with vector-specific primers to confirm amplification of correct targets.

### DNA manipulation

Coding sequences of different parasite genes were amplified by PCR from either genomic DNA or cDNA (indicated in Additional file 10) using *Pfu* DNA Polymerase (Thermo Scientific) according to manufacturer’s instructions. The gene-specific primers used for amplification and subsequent cloning into pET41a(+) are also listed in Additional file 10. All plasmids were sequenced with vector-specific primers to confirm correct insertion into pET41a(+).

### Recombinant protein expression

The pET41a(+) plasmid (Novagen®) was used for the expression of truncated forms of EtHOWP1 (residues 20–301 of ETH_00018895), EtHAP2 (75 residues coded by the genomic region 210518–210294 of HG674888), TgHOWP1 (residues 24–407 of TGME49_316890) and TgAO2 (residues 176–532 of TGME49_086780) proteins, producing recombinant proteins with N-terminal GST tags and flanking 6-His regions. An auto-induction protocol was followed for high-expression of GST-fusion proteins [62]. Briefly, plasmids were transformed into BL21 *Escherichia coli* and grown overnight in non-inducing, PG media at 37°C, 220 rpm. ZYP-5052 media was inoculated at 1 in 2,000 with overnight culture and grown for 20 h at 37°C, 220 rpm. A truncated form of EtSUB1 (residues 28–113 of ETH_00009790) and of a previous version of EtSUB2 (residues 456–685 of XM_001238654.1) were expressed with an C-terminal histidine tag in pET101/D-TOPO® (Invitrogen™, Australia) and a truncated form of EtAO2 (residues 44–1179 of ETH_00028385) was expressed with an N-terminal histidine tag in the E3 expression plasmid (GenScript USA Inc.) by standard IPTG induction in BL21 *Escherichia coli*. EtHOWP1-GST and TgHOWP1-GST were purified via their GST tags under native conditions using Glutathione Sepharose 4 Fast Flow (GE Healthcare) as per manufacturer’s instructions and eluted in 30 mM reduced GSH. EtHAP2-GST and TgAO2-GST were purified via their 6-His tags under denaturing conditions using NiNTA agarose (Qiagen) as per manufacturer’s instructions and eluted in 250 mM imidazole. EtSUB1, EtSUB2 and EtAO2 were purified via the 6-His tag under native conditions using the Profina^TM^ protein purification system and Native IMAC Purification Kit (Bio-Scale Mini Profinity IMAC nickel-chelating column and desalting column) as per manufacturer’s instructions. Purified proteins were dialysed overnight at 4°C in 25 mM ammonium acetate using a Slide-A-Lyzer Dialysis Cassette, 10 K molecular weight cut-off (Thermo Scientific) and stored at −20°C.

### Antibodies

Polyclonal sera was collected from mice immunised with recombinant forms of EtHOWP1-GST, EtHAP2-GST, TgHOWP1-GST, TgAO2-GST, EtSUB2 and EtAO2 using procedures described previously [30]. Polyclonal sera was also collected from rabbits immunised with a synthetic peptide (Auspep Pty. Ltd.) representing EtOWP6 (residues 164–179 of ETH_00012470) conjugated to KLH (keyhole limpet haemocyanin) carrier protein, using methods described previously [15]. Importantly, although the primary amino acid sequence of the recombinant EtSUB2 antigen is partially conserved with other *E. tenella* subtilisins, anti-EtSUB2 sera reactivity was shown by ELISA to be specific to the EtSUB2 antigen, not reacting significantly with any other subtilisin, including EtSUB1, with which it shares 37% identity. Briefly, for the assessment of antibody specificity by ELISA, recombinant protease antigens were diluted in ELISA buffer 1 to a final concentration of 5 ng/ml, and 100 ml aliquots (500 ng per well) added to a 96-well plate. Plates were incubated overnight at 4°C to allow the antigen to bind. The following day, plates were washed twice with PBS/0.05% TWEEN20® and once with PBS to remove unbound antigen. Plates were then blocked with 200 ml per well of PBS/5% SMP for 1 h at room temperature with gentle agitation. Plates were then washed as before, and 100 ml aliquots of primary antibody diluted in PBS/2.5% SMP added to each well and incubated for 1 h at room temperature with gentleagitation. The plates were washed again as above and 100 μL aliquots of secondary antibody (goat-derived, anti-rabbit or anti-mouse IgG, H&L chain-alkaline phosphatase conjugate) diluted at 1:2000 in PBS/2.5% SMP were added to eachwell. Plates were incubated for a further hour at room temperature, washed again and 200 μL of 1 mg/ml p-nitrophenyl phosphate added to each well. The reaction was allowed to develop for 20mins at 37°C, with a maximum of 60 min, and absorbance was read at 405 nm using the FLUOstar Omega® multi-detection microplate reader (BMG Labtech). All samples were tested in quadruplicate.

### Immunofluorescent microscopy

Sections of 3 μm were prepared from paraffin-embedded samples of *E. tenella*-infected chicken caeca (144 h p.i.) and *T. gondii*-infected cat intestine (day 7). Deparaffinisation, antigen retrieval and immunofluorescence analysis were carried out as described previously [17], using antibodies described in the Results section. Alternatively, unsporulated oocysts were vortexed with an equal volume of glass beads (710–1,180 μm, Sigma) and air-dried onto glass slides prior to immunofluorescence assays. Alexa Fluor® 488 goat anti-mouse (green) and Alexa Fluor® 594 goat anti-rabbit (red) secondary antibodies were used at a dilution of 1 in 500 for detecting mouse and rabbit primary antibodies, respectively, and DAPI counterstaining was used at 1 μg/ml. Samples were mounted in VECTASHIELD® Mounting Medium (Vector Laboratories). Epifluorescence imaging was performed using a Leica DMI 6000 B microscope and the Leica LAS AF software. Confocal imaging was performed using a Leica SP2 AOBS confocal laser-scanning microscope and the Leica Confocal software for data collection, with subsequent deconvolution using the Huygens Essential (Scientific Volume Imaging B.V.) software.

### Western blot

Protein samples were prepared from *E. tenella* and uninfected host caecal samples and analysed by Western blot under reducing and alkylating conditions as described previously [17]. Again, the initial lysis of oocyst samples was achieved by vortexing with glass beads (as described above). Protein concentration was determined using a NanoDrop ND-1000 spectrophotometer. Membranes were probed with either mouse or rabbit sera (at dilutions described in the results) and subsequently with goat anti-mouse or anti-rabbit IgG (heavy and light chain), horseradish peroxidase conjugates (Life technologies) diluted at 1 in 5,000. Probed membranes were developed using CPS-1 Chemiluminescent Peroxidase Substrate-1 (Sigma) according to manufacturer’s instructions. The membranes were visualised using the VersDocTM Imaging System (BioRad) and images analysed using the Quantity One® 1-D Analysis Software (BioRad).

### Gene annotation and sequences analysis

Where possible, existing gene annotations available on www.toxodb.org were used for *E. tenella* genes described in this study. For hypothetical genes, additional annotation was achieved through Blast2Go analysis (www.blast2go.com) of the predicted protein sequences, using a cut-off of E-Value < 0.0005. The identification of potential homologues of *E. tenella* proteins was also carried out using BlastP on the non-redundant NCBI database or on www.toxodb.org. Multiple alignments of protein and DNA sequences were performed using ClustalW (http://www.ebi.ac.uk/Tools/msa/clustalw2/). The prediction of signal peptides in protein sequences was carried out using the SignalP 4.1 Server (www.cbs.dtu.dk/services/SignalP/).

### Statistical analysis

One-way analysis of variance (ANOVA) were carried out on qRT-PCR results using GraphPad Prism® Version 6.03 (GraphPad Software Inc., USA).

### Ethics statement

Animal experiments performed in Australia were carried out according to the specific regulations of the Australian Code of Practice for the Care and Use of Animals for Scientific Purposes. Mouse experiments performed in Cairns (permit number A1812) were approved by the Animal Ethics Committee at James Cook University. Chicken experiments performed in Sydney were approved by the University of Technology Animal Care and Ethics Committee (protocols 2008–096 and 2008–188). Animal experiments performed in Zurich, Switzerland were approved by the Veterinary Office and the Ethics Committee of the Canton of Zurich (Kantonales Veterinaramt Zurich, Obstgartenstrasse 21, 8090 Zurich, Switzerland) and carried out according to Swiss law and guidelines on Animal Welfare and the specific regulations of the Canton of Zurich. Permit number 171/2013 covers the production of polyclonal sera in mice, while permit number 108/2010 covers all mouse and cat experiments required for *T. gondii* sexual stage propagation. Experiments performed in Tours, France (protocol registration 2012-11-9) were approved by the ethics committee CEEA VdL and were carried out according to French legislation (French Government Decree 2001–464) and EEC regulations (86/609/CEE).

## Additional files

Additional file 1:
**Sequencing the** ***E. tenella*** **gametocyte transcriptome using RNA Seq.** (**a**, **b**) A gametocyte sample purified from *E. tenella*-infected chickens at 144 h post-infection viewed under bright-field **(a)** or after DAPI staining **(b)**. DAPI stains the nuclei of multiple microgametes and faintly stains the nuclei of a single macrogametocyte. Scale = 10 μm. **(c)** Total RNA extracted from triplicate samples of *E. tenella* gametocytes (Ga, Gb, Gc), merozoites (Ma, Mb, Mc) and sporozoites (Sa, Sb, Sc) was analysed using a Bioanalyzer 2100 (Agilent). Parasite large ribosomal RNA bands, 26S and 18S, are detected in all samples, while a faint, host-specific 28S ribosomal RNA band is detected in the gametocytes samples only (arrow). **(d)** The number of total paired reads generated for each of the nine RNA Seq experiments is plotted alongside the number of reads mapping uniquely to exon models of *E. tenella*. Additional file 2:
**Total paired reads mapped uniquely to predicted exon models of** ***E. tenella.*** Listed for each of the nine RNA Seq libraries are the total number of paired reads uniquely mapped to the predicted exon model of each of the 8,786 *E. tenella* genes. The nine RNA Seq libraries were produced from triplicate samples of *E. tenella* gametocytes (Ga, Gb, Gc), merozoites (Ma, Mb, Mc) and sporozoites (Sa, Sb, Sc). Additional file 3:
**Differential expression of gene transcription in** ***E. tenella*** **gametocytes.** Listed for each of the 8,786 *E. tenella* genes are the normalised counts calculated by DESeq in each of the three developmental stages (gametocytes, merozoites and sporozoites), along with the calculated differential expression in gametocytes over either merozoites or sporozoites. The fold change, log2 fold change, p-value and adjusted p-value are also listed. ‘Max’ indicates where transcript was exclusively detected in gametocytes, ‘Min’ indicates where transcript was exclusively detected in the reference asexual stage and ‘NA’ indicates where transcript was not detected in either stage. Additional file 4:
**Transcript abundance and annotation of** ***E. tenella*** **upregulated gametocyte transcripts.** Listed for each of the 863 upregulated *E. tenella* gametocyte transcripts is the publicly available gene annotation (www.toxodb.org), an annotation derived manually from Blast2Go analysis and the manually assigned biological function. As a measure of transcript abundance, the FPKM (Fragments Per Kilobase of exon model per Million mapped reads) of each gene is listed for each of the three developmental stages of *E. tenella*. Additional file 5:
**Sequence alignment of ETH_00019840 and ETH_00019845 proteins.** The amino acid sequences of ETH_00019840 and ETH_00019845, coded by the first and second most abundant upregulated *E. tenella* gametocyte transcripts (respectively), were aligned using ClustalW. ‘*’ indicates the alignment of identical amino acids, while ‘:’ and ‘.’ indicate the alignment of conserved and partially conserved amino acids. Additional file 6:
**Sequence alignment of ETH_00019835 and ETH_00032470 proteins.** The amino acid sequences of ETH_00019835 and ETH_00032470, coded by the third and fourth most abundant upregulated *E. tenella* gametocyte transcripts, were aligned using ClustalW. ‘*’ indicates the alignment of identical amino acids, while ‘:’ and ‘.’ indicate the alignment of conserved and partially conserved amino acids. Additional file 7:
**Conservation of cysteine-rich oocyst wall proteins.** The amino acid sequences of EtOWP6 (ETH_00012470), TgOWP6 (TGME49_286250) and CpOWP6 (cgd4_3090) were aligned using ClustalW. The position of cysteine residues which mark the beginning of cysteine-rich Type I repeats are indicated with arrows. ‘*’ indicates the alignment of identical amino acids, while ‘:’ and ‘.’ indicate the alignment of conserved and partially conserved amino acids. Additional file 8:
**Conservation of Hypothetical Oocyst Wall Protein 1 (HOWP1) in** ***E. tenella*** **and** ***T. gondii.*** The amino acid sequences of EtHOWP1 (ETH_00018895) and TgHOWP1 (TGME49_316890) were aligned using ClustalW. ‘*’ indicates the alignment of identical amino acids, while ‘:’ and ‘.’ indicate the alignment of conserved and partially conserved amino acids. Additional file 9
**Conservation of Amine Oxidase 2 (AO2) in** ***E. tenella*** **and** ***T. gondii.*** The amino acid sequences of (i) the MAM domain and (ii) the Cu + amine oxidase enzyme domain of EtAO2 (ETH_00028385) and TgAO2 (TGME49_086780) were aligned using ClustalW. The MAM domain corresponds to residues 235–408 and 176–347 of EtAO2 and TgAO2, respectively. The Cu + amine oxidase enzyme domain corresponds to residues 1,178-1,612 and 1,109-1,505 of EtAO2 and TgAO2, respectively. ‘*’ indicates the alignment of identical amino acids, while ‘:’ and ‘.’ indicate the alignment of conserved and partially conserved amino acids. Additional file 10:
**Primers used in this study.** The name, sequence and direction of each primer used throughout this study are listed. Also provided are the target gene name/annotation, template source for PCR and purpose of amplification. Additional comments are also provided.

## Abbreviations

FPKM: Fragments per kilobase of exon model per million mapped reads
CDS: Coding sequence
DE: Differential expression
WFB: Wall forming bodies
HOWP1: Hypothetical oocyst wall protein 1
GCS1-HAP2: Generative cell specific 1-HAPLESS 2
