# Supplementary figures and images for: RNA Seq analysis of the Eimeria tenella gametocyte transcriptome reveals clues about the molecular basis for sexual reproduction and oocyst biogenesis
Source: BMC Genomics. 2015 Feb 18;16(1):94. doi: 10.1186/s12864-015-1298-6 (PMC4345034; doi:10.1186/s12864-015-1298-6)

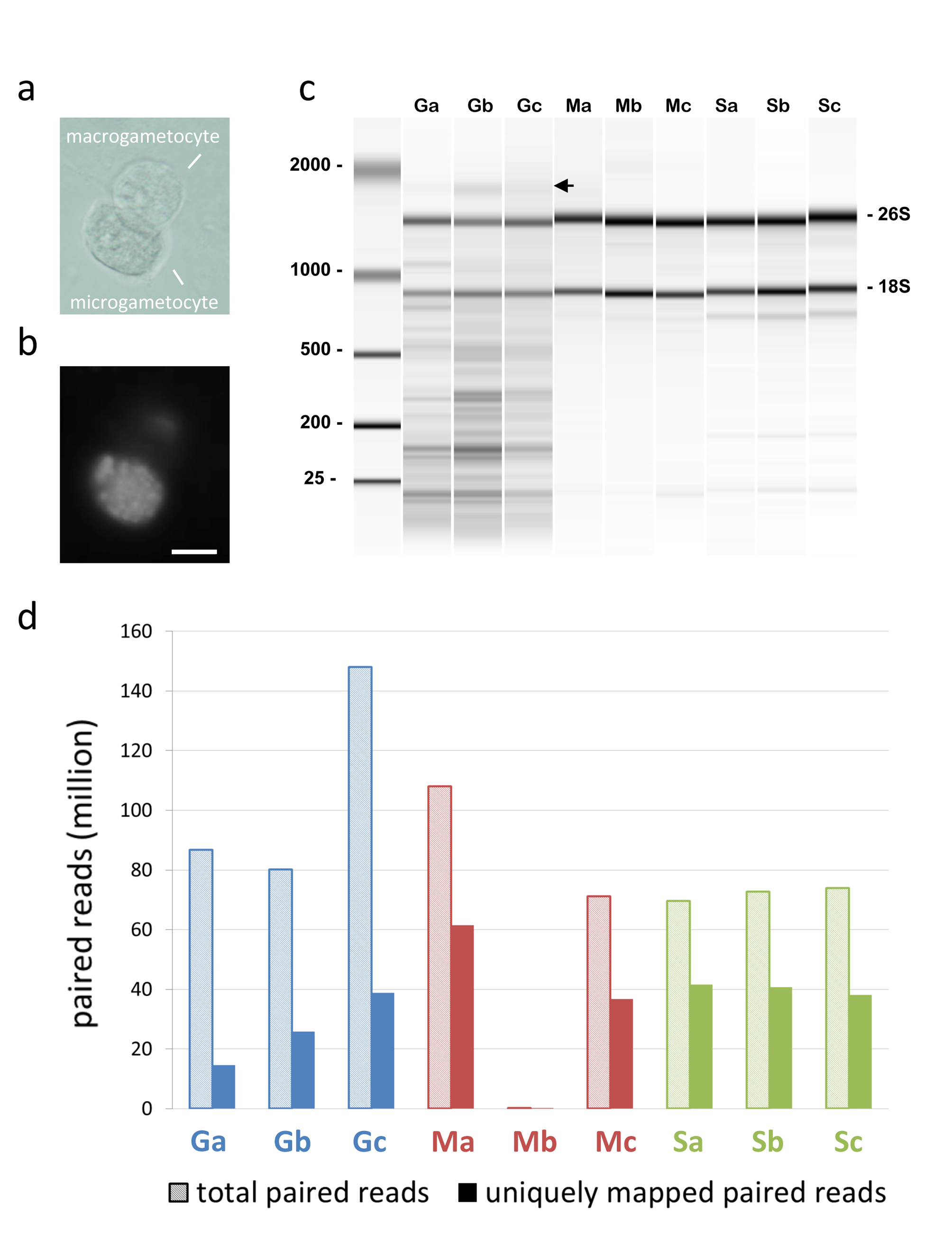

Supplement: Additional file 1: — Sequencing the E. tenella gametocyte transcriptome using RNA Seq. (a, b) A gametocyte sample purified from E. tenella-infected chickens at 144 h post-infection viewed under bright-field (a) or after DAPI staining (b). DAPI stains the nuclei of multiple microgametes and faintly stains the nuclei of a single macrogametocyte. Scale = 10 μm. (c) Total RNA extracted from triplicate samples of E. tenella gametocytes (Ga, Gb, Gc), merozoites (Ma, Mb, Mc) and sporozoites (Sa, Sb, Sc) was analysed using a Bioanalyzer 2100 (Agilent). Parasite large ribosomal RNA bands, 26S and 18S, are detected in all samples, while a faint, host-specific 28S ribosomal RNA band is detected in the gametocytes samples only (arrow). (d) The number of total paired reads generated for each of the nine RNA Seq experiments is plotted alongside the number of reads mapping uniquely to exon models of E. tenella. [file 12864_2015_1298_MOESM1_ESM.tiff]

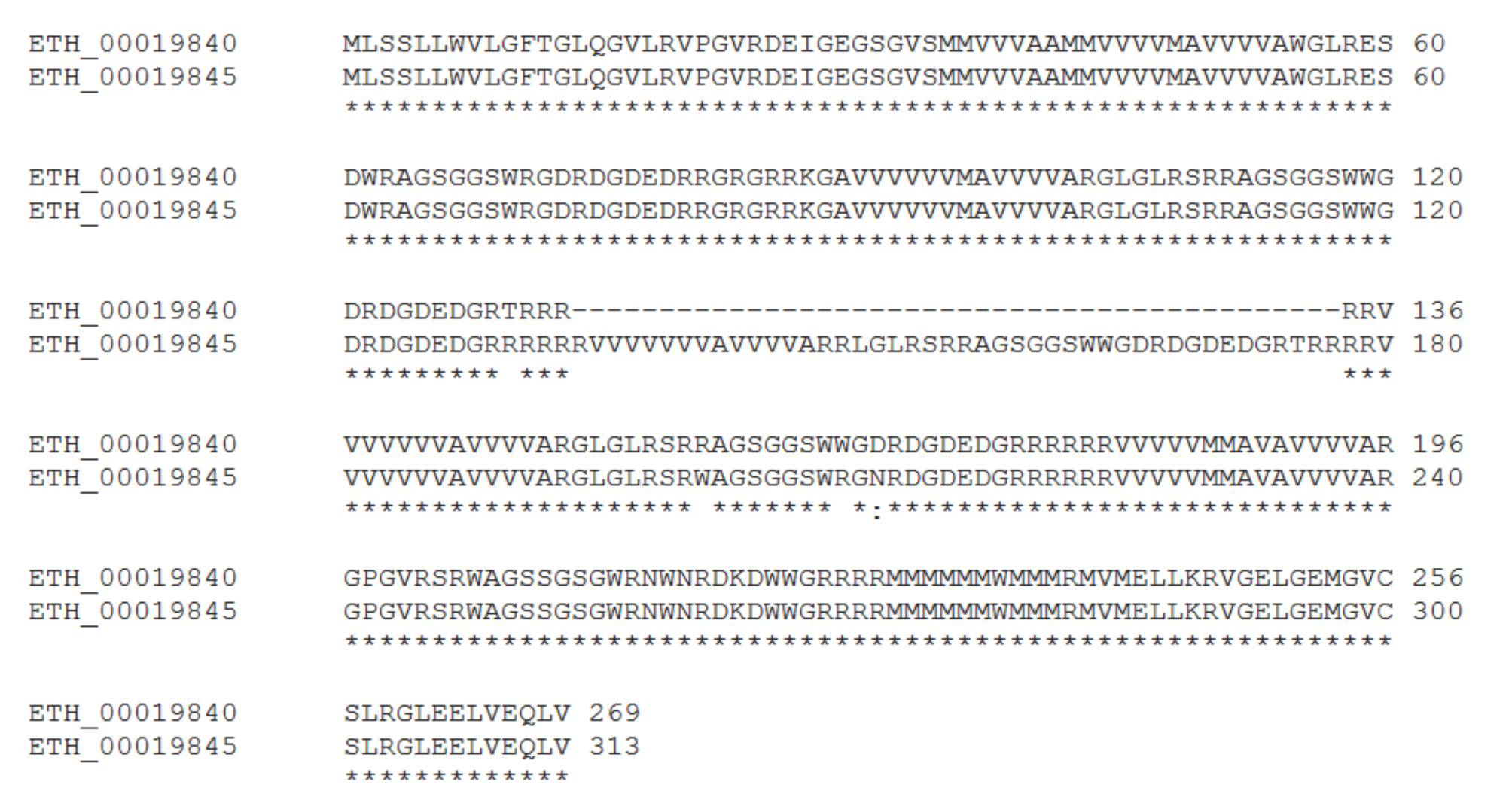

Supplement: Additional file 5: — Sequence alignment of ETH_00019840 and ETH_00019845 proteins. The amino acid sequences of ETH_00019840 and ETH_00019845, coded by the first and second most abundant upregulated E. tenella gametocyte transcripts (respectively), were aligned using ClustalW. ‘*’ indicates the alignment of identical amino acids, while ‘:’ and ‘.’ indicate the alignment of conserved and partially conserved amino acids. [file 12864_2015_1298_MOESM5_ESM.tiff]

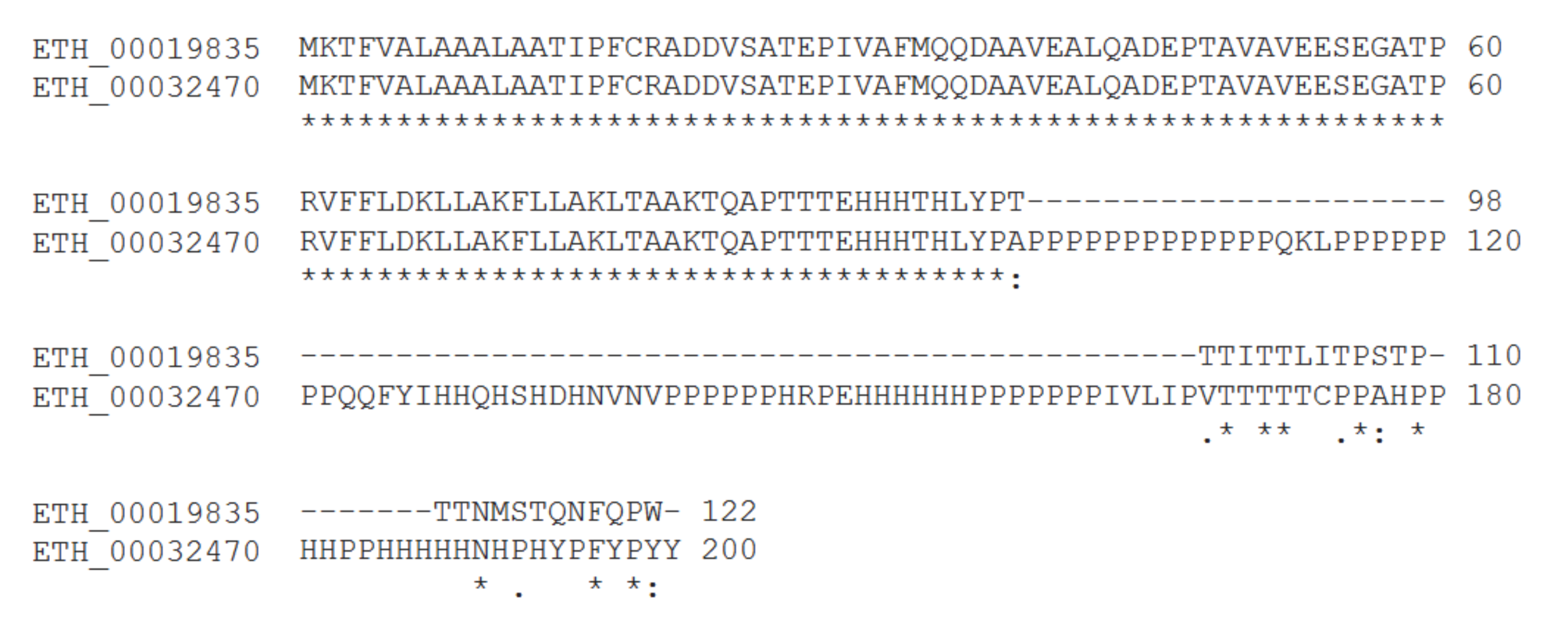

Supplement: Additional file 6: — Sequence alignment of ETH_00019835 and ETH_00032470 proteins. The amino acid sequences of ETH_00019835 and ETH_00032470, coded by the third and fourth most abundant upregulated E. tenella gametocyte transcripts, were aligned using ClustalW. ‘*’ indicates the alignment of identical amino acids, while ‘:’ and ‘.’ indicate the alignment of conserved and partially conserved amino acids. [file 12864_2015_1298_MOESM6_ESM.tiff]

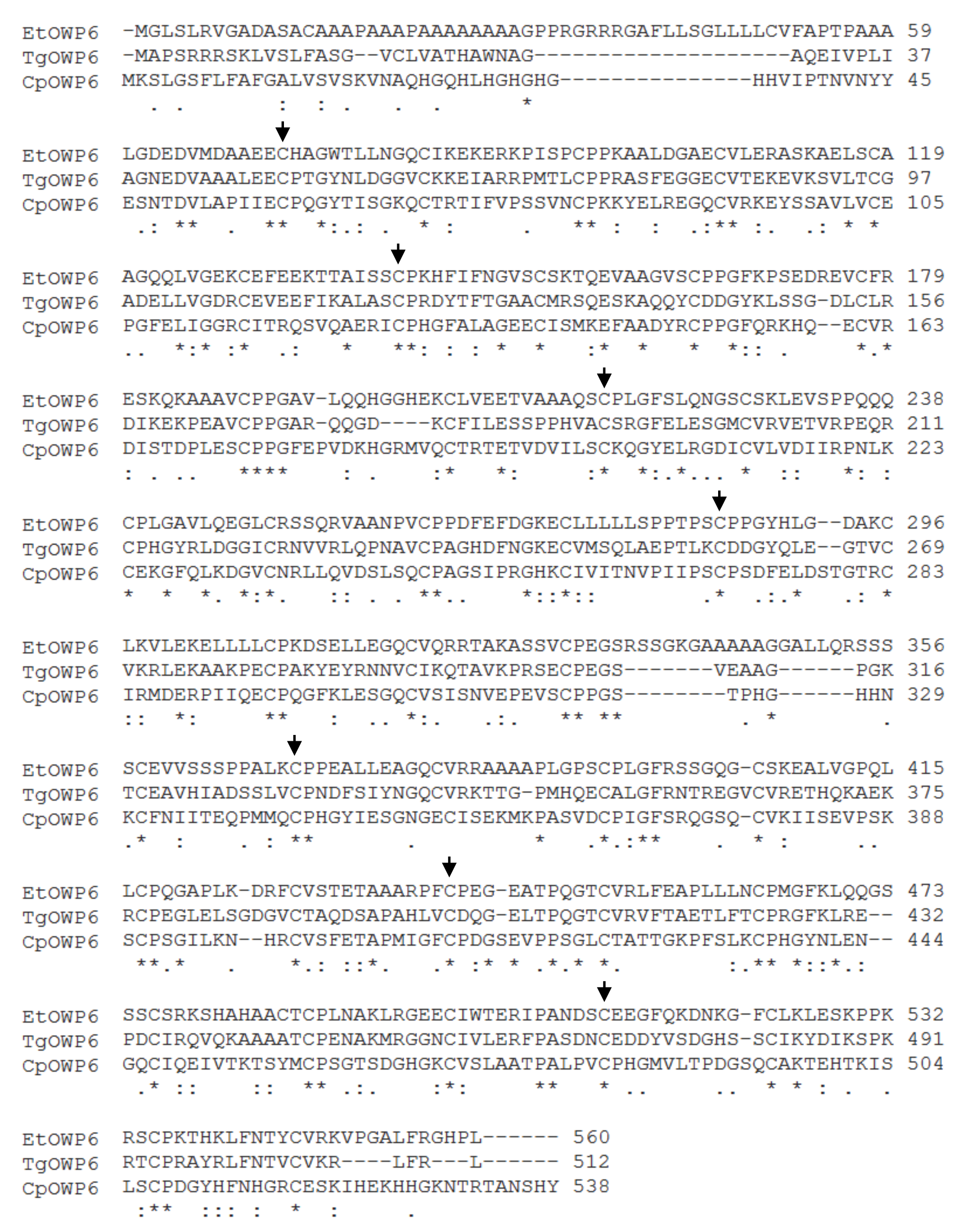

Supplement: Additional file 7: — Conservation of cysteine-rich oocyst wall proteins. The amino acid sequences of EtOWP6 (ETH_00012470), TgOWP6 (TGME49_286250) and CpOWP6 (cgd4_3090) were aligned using ClustalW. The position of cysteine residues which mark the beginning of cysteine-rich Type I repeats are indicated with arrows. ‘*’ indicates the alignment of identical amino acids, while ‘:’ and ‘.’ indicate the alignment of conserved and partially conserved amino acids. [file 12864_2015_1298_MOESM7_ESM.tiff]

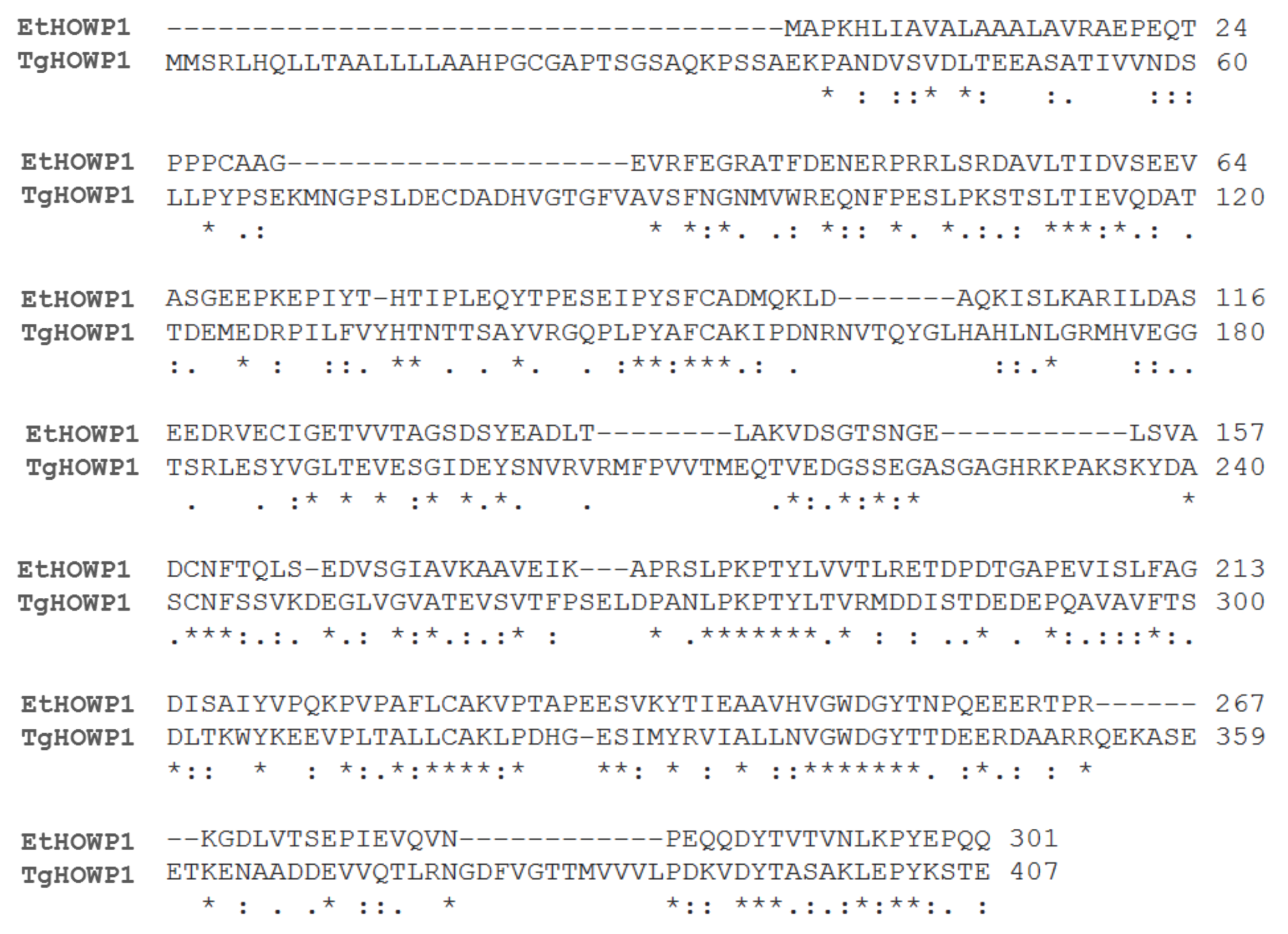

Supplement: Additional file 8: — Conservation of Hypothetical Oocyst Wall Protein 1 (HOWP1) in E. tenella and T. gondii. The amino acid sequences of EtHOWP1 (ETH_00018895) and TgHOWP1 (TGME49_316890) were aligned using ClustalW. ‘*’ indicates the alignment of identical amino acids, while ‘:’ and ‘.’ indicate the alignment of conserved and partially conserved amino acids. [file 12864_2015_1298_MOESM8_ESM.tiff]

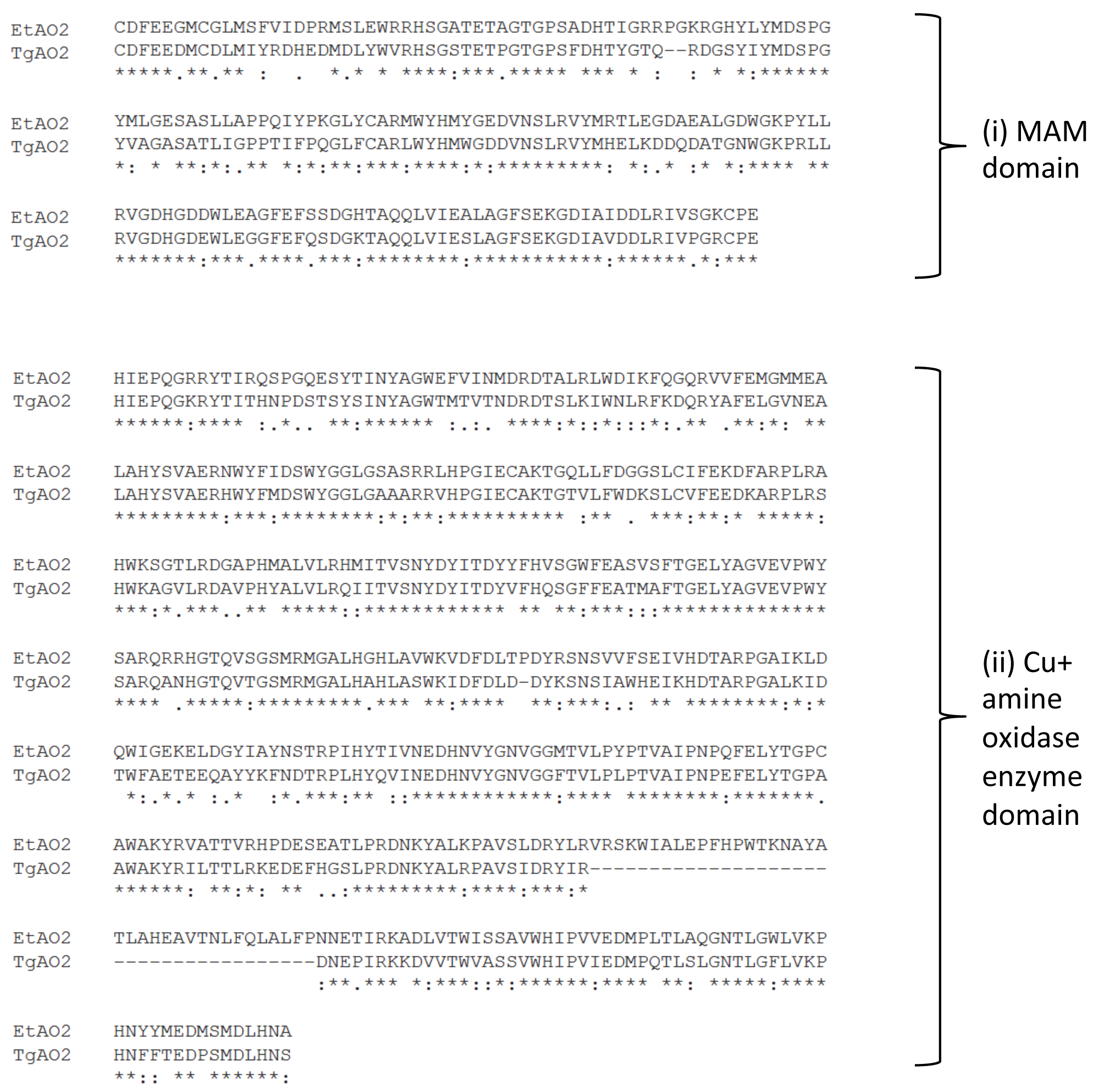

Supplement: Additional file 9 — Conservation of Amine Oxidase 2 (AO2) in E. tenella and T. gondii. The amino acid sequences of (i) the MAM domain and (ii) the Cu + amine oxidase enzyme domain of EtAO2 (ETH_00028385) and TgAO2 (TGME49_086780) were aligned using ClustalW. The MAM domain corresponds to residues 235–408 and 176–347 of EtAO2 and TgAO2, respectively. The Cu + amine oxidase enzyme domain corresponds to residues 1,178-1,612 and 1,109-1,505 of EtAO2 and TgAO2, respectively. ‘*’ indicates the alignment of identical amino acids, while ‘:’ and ‘.’ indicate the alignment of conserved and partially conserved amino acids. [file 12864_2015_1298_MOESM9_ESM.tiff]
